# On the Question of the Regio-, Stereoselectivity and the Molecular Mechanism of the (3+2) Cycloaddition Reaction Between (*Z*)-C-Phenyl-N-alkyl(phenyl)nitrones and (*E*)-3-(Methylsulfonyl)-propenoic Acid Derivatives

**DOI:** 10.3390/molecules30244738

**Published:** 2025-12-11

**Authors:** Martyna Ząbkowska, Karolina Kula, Volodymyr Diychuk, Radomir Jasiński

**Affiliations:** 1 Cracow University of Technology, Faculty of Chemical Engineering and Technology, Warszawska 24, 31-155 Cracow, Poland; martyna.zabkowska@student.pk.edu.pl; 2 Yuriy Fedkovych Chernivtsi National University, Department of Chemistry and Food Examination, Kotsiubynskoho 2, 58-002 Chernivtsi, Ukraine; diychuk@chnu.edu.ua

**Keywords:** (3+2) cycloaddition, nitrones, isoxazolidines, regioselectivity, stereoselectivity, molecular mechanism, DFT, MEDT, in silico

## Abstract

In this work, the regio- and stereochemistry as well as the molecular mechanism of the cycloaddition reaction of nitrones with (*E*)-3-(methylsulfonyl)-propenoic acid derivatives were analyzed based on ωb97xD/6-311G(d,p) quantum chemical calculations. In light of these data, it is possible to propose selectivity of the analyzed processes, which was not clearly determined in light of previous experimental studies. Furthermore, the mechanism of the process was diagnosed. CDFT descriptors indicate that the reaction is triggered by a nucleophilic attack of the nitrone oxygen atom on the electrophilic carbon atom of (*E*)-3-(methylsulfonyl)-propenoic acid derivatives. In turn, PES analysis shows that, despite the nucleophilic-electrophilic character of the reactants, the corresponding transition states are only weakly polar and highly synchronous. IRC calculations rule out zwitterionic or biradical intermediates, confirming a single-step mechanism. The in silico ADME and PASS predictions indicate that the resulting isoxazolidines possess promising biological profiles, showing potential modulation of the serotonin system through 5-HT2A and 5-HT2C antagonism and stimulation of serotonin release, with structural features compatible with P450-mediated metabolism. Considering this attractive application potential, a detailed mechanistic investigation of their formation becomes essential for understanding and ultimately controlling the reaction pathways leading to these heterocycles.

## 1. Introduction

Isoxazolidines (tetrahydroisoxazoles) are an important group of building blocks used in modern chemical technology, biotechnology and pharmaceutical research [1,2]. They occur in numerous functional materials and bioactive structures. In particular, isoxazoline scaffolds have attracted growing interest in agrochemical research, where they serve as structural motifs in herbicides, insecticides and fungicides [3,4]. At the same time, isoxazolidine frameworks remain widely exploited for the design of compounds with promising pharmacological potential, such as antitumor agents [5,6], antibacterial substances [7,8], anti-inflammatory agents [9,10] and many other biologically active molecules [11,12,13]. Isoxazolidines containing good leaving groups (LGs) [14] are often prone to ring transformation into β-lactams [15,16,17], which form a well-known molecular core of many clinically significant antibiotics [18,19,20].

The most universal strategy for the synthesis of isoxazolidines relies on (3+2) cycloaddition (32CA) reactions of nitrones [21,22,23]. This approach benefits from the broad availability of three-atom components (TACs) and a wide variety of suitable alkenes [24,25], which together create a highly flexible synthetic platform. The method offers excellent atom economy, reaching 100 percent under ideal conditions [26,27], and provides access to a broad spectrum of heterocycles. A central advantage of nitrone cycloaddition chemistry is the high level of stereocontrol that can be achieved in these reactions. Both the configuration of the nitrone and the geometry of the alkene strongly influence the relative and absolute stereochemistry of the resulting isoxazolidines [28,29,30]. This feature is particularly valuable for applications in medicinal chemistry, where precise spatial arrangement often determines biological activity [31]. Advances in catalytic and asymmetric variants of nitrone cycloadditions have further expanded the stereochemical versatility of this methodology [32,33].

**Scheme 1:**
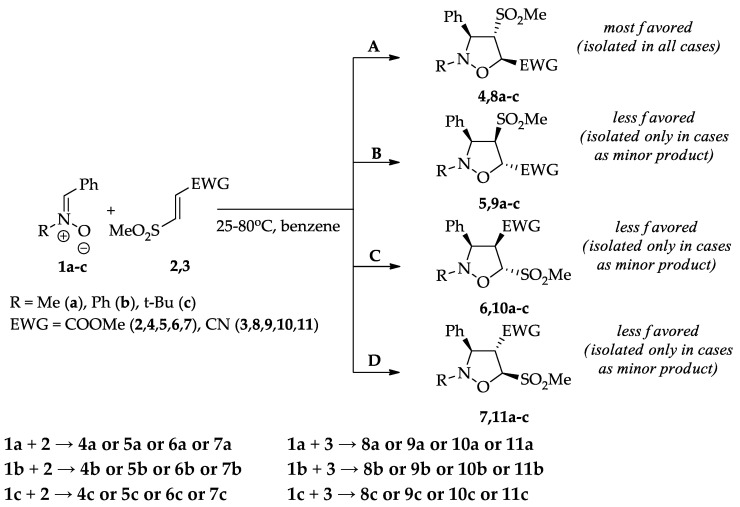
Theoretically possible pathways of 32CA reactions of (*Z*)-C-phenyl-N-alkyl(phenyl)-nitrones (**1a**–**c**) with (*E*)-3-(methylsulfonyl)-propenoic acid derivatives (**2** and **3**).

Some years ago, *Chanet-Ray* and coworkers [34] experimentally investigated the 32CA between (*Z*)-C-phenyl-N-methylnitrone (**1**) and the methyl ester of (*E*)-3-(methylsulfonyl)-propenoic acid (**2**). They reported that this reaction could proceed under mild conditions, affording a mixture of three out of the four theoretically possible regio- and stereoisomeric 2-methyl-3-phenylisoxazolidines. Similarly, a number of other reactions involving nitrones and (*E*)-3-(methylsulfonyl)-propenoic acid derivatives (**1a**–**c** + **2** or **3**) were analyzed (Scheme 1).

However, the account of these transformations in their paper is incomplete and includes several omissions and ambiguities. In particular, the following points require further clarification:

**(i) Regio- and stereochemistry.** The regio- and stereochemical outcome of the reaction remains unclear. The authors only partially separated the post-reaction mixture and inferred the product constitution from partial NMR data recorded on a low-frequency instrument. The only information available indicates that, in each case, the most favored product should be the corresponding 3,4-*trans*-3-phenyl-4-methylsulfonyl-isoxazolidine (**4** or **8**). Notably, HPLC and NMR analyses of the crude reaction mixtures, which are standard practices for assessing regio- and stereoselectivity in 32CA reactions [35,36,37], were never performed. Consequently, it is unknown whether other regio- and stereoisomeric adducts were formed, particularly since the yield of isolated products was sometimes as low as a few percent.

**(ii) Reaction selectivity.** The selectivity of the reaction is not fully interpreted. As a result, no reliable conclusions can be drawn regarding the likely selectivity of analogous reactions involving alkylsulfonyl-substituted alkenes.

**(iii) Reaction mechanism.** The mechanism of the reaction remains undetermined. Until about 30 years ago, all 32CA reactions were generally assumed to proceed via a single-step “concerted” mechanism [38,39,40]. Current knowledge, however, indicates that some 32CA reactions proceed via a two-step pathway involving either a zwitterionic [41,42,43] or a diradical intermediate [44,45,46]. Moreover, even in single-step reactions, electron density reorganization does not necessarily occur in a purely pericyclic manner; rather, it involves a complex sequence of stages, with multiple directions and modes of electron density transfer between molecular fragments [47,48,49,50].

Considering the issues outlined above, we carried out a comprehensive quantum-chemical investigation of the studied reaction using both the Conceptual Density Functional Theory (CDFT) [51,52,53] and the Molecular Electron Density Theory (MEDT) [24]. Our goal was to obtain a complete, mechanistically grounded picture of the factors controlling its reactivity and selectivity. First, we examined the electronic structures of all reactants, identifying the most electrophilic and nucleophilic reactive centers and clarifying their roles in the initial stage of the cycloaddition. We then simulated all theoretically feasible reaction pathways, which enabled us to characterize their mechanisms, energetic profiles, and the factors governing regio- and stereoselectivity. Finally, we analyzed the reorganization of the electron density along the reaction coordinate of the model process to determine which electronic rearrangements dictate the course of the reaction and the stability of the associated intermediates and products.

Additionally, we performed preliminary in silico studies to evaluate the potential biological activity of the cycloaddition products (**4**–**11**). This approach is particularly important because the biological activity of these isoxazolidines has not yet been studied experimentally. No activity has been reported, and no attempts have been made to identify them as potential APIs. These analyses employed both ADME (Absorption, Distribution, Metabolism, and Excretion) [54] and PASS (Prediction of Activity Spectra for Substances) [55] predictions to assess pharmacokinetic parameters, drug-likeness, and possible activity spectra. The results provide initial but promising evidence for the pharmacological relevance of these compounds and complement the mechanistic investigations presented in this work. Altogether, the combination of detailed mechanistic insights and bioactivity predictions offers a coherent understanding of the reactivity and functional significance of the resulting isoxazolidines. This integrated approach offers a clear basis for understanding the factors that influence the formation and stability of sulfonyl-substituted isoxazolidines in 32CA reactions and helps place their potential biological relevance in a broader synthetic context. Approaches of this kind are increasingly used to relate mechanistic features to functional properties, making them a practical and well-established element of modern studies on cycloaddition processes [56,57]. We believe that this aspect significantly strengthens the study and enhances its potential interest to the research community.

## 2. Results and Discussion

### 2.1. Analysis of the Reaction Mechanism

First, we decided to examine the mechanistic aspects of the studied 32CA reaction in a systematic and comprehensive manner. To achieve this, we began with an in-depth analysis of the electronic structures of the reacting substrates within the framework of CDFT. This approach allowed us to identify the most reactive centers, assess polarity patterns, and anticipate the general reactivity profile expected for the cycloaddition. After establishing the electronic characteristics of both partners, we proceeded to investigate the full reaction pathway by exploring the potential energy surface (PES). This analysis made it possible to evaluate the energetic feasibility of the individual pathways, determine the origin of regio- and stereochemical preferences, and clarify the fundamental factors governing the reactivity of this system.

#### 2.1.1. Global and Local Interactions Between Reactants

CDFT is one of the essential approaches for analyzing the reactivity of molecules in polar reactions. This framework links classical chemical descriptors, such as electronic chemical potential (μ) as well as chemical hardness (η), with the electronic structure of molecules. Using these parameters, one can determine global parameters, including electrophilicity (ω) and nucleophilicity (N), which help assign the role of each reactant as either an electrophile or a nucleophile in a given reaction [58,59]. Such information is particularly important for predicting which regioisomers are likely to form [60,61]. Thus, the global reactivity indices of (*Z*)-C-phenyl-N-alkyl(phenyl)nitrones (**1a**–**c**) and (*E*)-3-(methylsulfonyl)-propenoic acid derivatives (**2** and **3**) are presented in Table 1.

**Figure 1:**
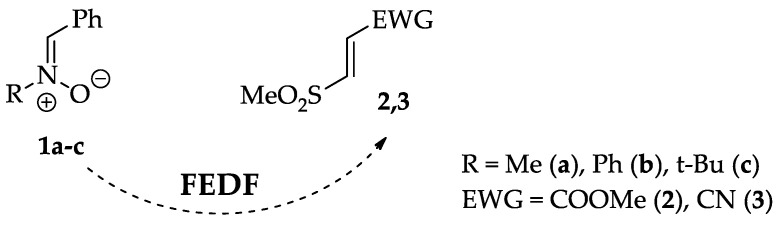
Visualization of electron density flux from nitrones (**1a**–**c**) to (*E*)-3-(methylsulfonyl)-propenoic acid derivatives (**2**–**3**) for tested 32CAs.

The applicability of CDFT depends strongly on the polarity of the studied processes [62,63], as this approach is valid only for polar reactions. Therefore, a preliminary assessment of the reaction polarity is required before any further analysis. To this end, the electronic chemical potential μ was examined. Thus, the electronic chemical potential μ of the (*Z*)-C-phenyl-N-alkyl(phenyl)nitrones (**1a**–**c**) is markedly higher than that of the corresponding (*E*)-3-(methylsulfonyl)-propenoic acid derivatives (**2** and **3**) (Table 1). This difference indicates that the electron density will flow from nitrones (**1a**–**c**) toward ethenes (**2** and **3**) (Figure 1). Consequently, the reaction between these reagents can be classified as a forward electron density flux (FEDF) process [64]. Additionally, the difference in global electrophilicity between nitrones (**1a**–**c**) and (*E*)-3-(methylsulfonyl)-propenoic acid derivatives (**2** and **3**) suggests that the investigated 32CAs exhibit only weak polar character (Table 1).

Subsequently, reactivity descriptors allowed us to assign the electrophilic and nucleophilic nature of the studied reagents. The computed global electrophilicity (ω) values [58] of the (*Z*)-C-phenyl-N-alkyl(phenyl)nitrones (**1a**–**c**) are 0.84 eV for **1a** (-Me), 0.80 eV for **1b** (-Ph), and 1.05 eV for **1c** (-t-Bu) (Table 1). The calculated global nucleophilicity (N) values [59] for these compounds are 3.73 eV for **1a** (-Me), 3.79 eV for **1b** (-Ph), and 3.75 eV for **1c** (-t-Bu) (Table 1). These values clearly indicate that all examined nitrones (**1a**–**c**) behave as moderate electrophiles while simultaneously acting as strong nucleophilic agents.

In turn, for both (*E*)-3-(methylsulfonyl)-propenoic acid derivatives (**2** and **3**), the computed global electrophilicity (ω) values [58] are 1.75 eV and 1.49 eV, respectively (Table 1). These results suggest that both ethenes (**2** and **3**) behave as strong electrophiles, with a considerably higher electrophilic character compared to the examined nitrones (**1a**–**c**). The computed global nucleophilicity (N) values [59] are 0.57 eV and 1.16 eV, respectively (Table 1). These results indicate that both ethenes (**2** and **3**) exhibit only marginal nucleophilic character, with global nucleophilicity values drastically lower than those of the examined nitrones (**1a**–**c**).

**Figure 2:**
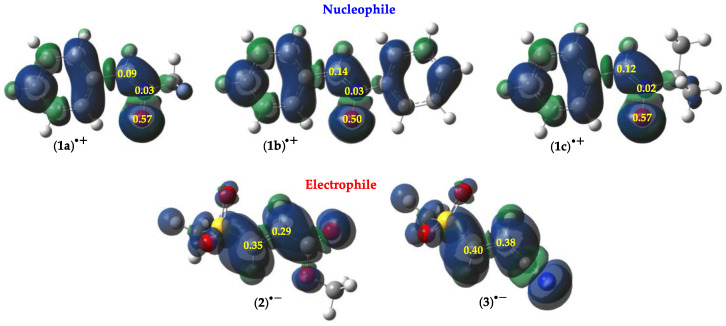
ωb97xD/6-311G(d,p) local electronic properties presented as three-dimensional atomic-spin representations of Mulliken densities for radical cations of nitrones (**1a**–**c**) and the radical anion of ethenes (**2**–**3**), together with the nucleophilic P_k_^−^ and the electrophilic P_k_^+^ Parr functions’ values.

Overall, in all the considered reactions, it can be concluded that the (*Z*)-C-phenyl-N-alkyl(phenyl)nitrones (**1a**–**c**) act as nucleophilic components, whereas the (*E*)-3-(methylsulfonyl)-propenoic acid derivatives (**2** and **3**) serve as electrophilic agents. This observation provides valuable guidance for predicting local reactivity in these systems. In reactions involving non-symmetric reagents, regioselectivity is typically governed by the interaction between the most electrophilic site of the electrophile and the most nucleophilic site of the nucleophile [65,66]. To identify these reactive centers, the electrophilic Pk^+^ and nucleophilic Pk^−^ Parr functions were computed, allowing the localization of the most electrophilic positions in ethenes (**2** and **3**) and the most nucleophilic positions in nitrones (**1a**–**e**) (Figure 2).

The analysis of the nucleophilic Pk^−^ Parr functions for the (*Z*)-C-phenyl-N-alkyl(phenyl)nitrones (**1a**–**c**) indicates that, in all cases and irrespective of the alkyl substituent, the oxygen atom constitutes the most nucleophilic center of the molecule. The values for P_N_^−^ are 0.57 for **1a** (-Me), 0.50 for **1b** (-Ph), and 0.57 for **1c** (-t-Bu) (Figure 2). In turn, the analysis of the electrophilic Pk^+^ Parr functions for the (*E*)-3-(methylsulfonyl)-propenoic acid derivatives (**2** and **3**) shows that both vinyl carbon atoms display comparable electrophilic characteristics. However, the carbon atom bonded to the methylsulfonyl group is consistently the more electrophilic site. Its Pk^+^ values amount to 0.35 for **2** (-COOMe) and 0.40 for **3** (-CN) (Figure 2). By contrast, the carbon atom connected to the EWG substituent exhibits lower electrophilicity, with Pk^+^ values of 0.29 for **2** (-COOMe) and 0.38 for **3** (-CN) (Figure 2).

**Figure 3:**
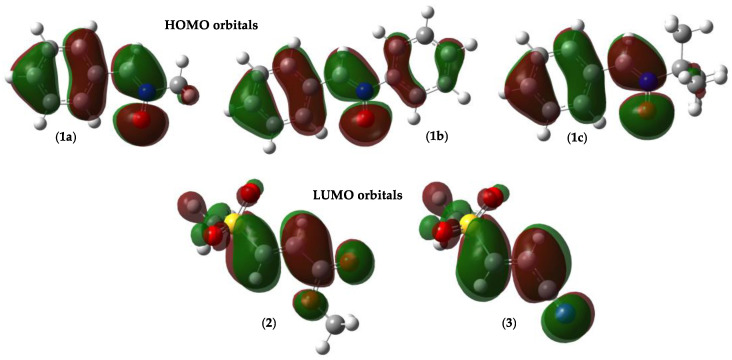
ωb97xD/6-311G(d,p) FMO visualization, showing the HOMO orbitals for nitrones (**1a**–**c**) and the LUMO orbitals for ethenes (**2**–**3**).

Analysis of intermolecular interactions, based on the older but still sometimes used Frontier Molecular Orbitals (FMO) theory [38,39], leads to similar conclusions. For all investigated reactions, the energy gap between the HOMO level of all nitrones (**1a**–**c**) and the LUMO level of the tested alkenes (**2** and **3**) are markedly smaller than the alternative gap, that is, between these alkenes (**2** and **3**) HOMO and nitrones (**1a**–**c**) LUMO (Table 1). This indicates that the discussed processes proceed under so-called normal orbital control [40], with the reaction course governed by electron transfer from the nitrone to the alkene. Examination of the local orbital function distributions shows that the process is consistently initiated by a nucleophilic attack of the oxygen atom within the CNO fragment of the nitrone. This arises from the fact that this atom exhibits a markedly higher HOMO density than the remaining atoms of the nitrone fragment, making it the dominant nucleophilic center (Figure 3). Mentioned nucleophilic attack may occur at either of the electrophilically activated centers of the alkene, as both corresponding LUMOs exhibit comparable shape and amplitude, providing similarly accessible sites for electron transfer (Figure 3).

**Figure 4:**
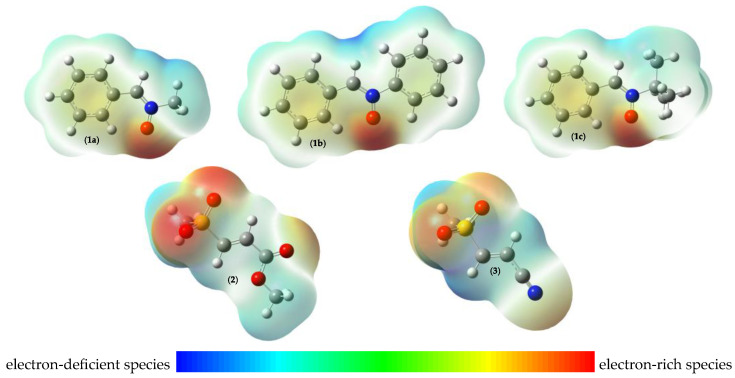
ωb97xD/6-311G(d,p) MEP maps for nitrones (**1a**–**c**) and ethenes (**2**–**3**). Regions of most negative (electron-rich) electrostatic potential are shown in red, whereas regions of most positive (electron-deficient) potential are shown in blue.

The presented results correlate well with the molecular electrostatic potential (MEP). In particular, the analysis of the MEP surfaces for all (Z)-C-phenyl-N-alkyl(phenyl)-nitrones (**1a**–**c**) reveals a region of highly negative electrostatic potential around the oxygen atom (shown in red) (Figure 4). This indicates that all studied nitrones (**1a**–**c**) act as nucleophiles, with the oxygen atom being the primary site for electron donation in the reaction. On the other hand, the both vinyl carbon atoms in (E)-3-(methylsulfonyl)propenoic acid derivatives (**2** and **3**) exhibit a slightly negative electrostatic potential (shown in light green) (Figure 4), indicating that these centers are equivalent and can act as electrophilic sites in the cycloaddition reaction.

#### 2.1.2. PES Exploration and Characterization of Critical Structures in the Studied CAs

Having established the general nature of the intermolecular interactions governing the studied processes, we next examined in detail the critical structures that define their reaction pathways. Accordingly, we undertook the systematic localization and characterization of all relevant transition states as well as any potential intermediates. This analysis was carried out using a computational protocol that has been extensively validated in previous theoretical investigations of various cycloaddition reactions [67,68,69], including numerous studies focused on nitrone-based systems [70,71]. The outcomes of these calculations, encompassing the structural features, energetics, and diagnostic reactivity parameters of the key stationary points, are comprehensively summarized in Table 2 and Table 3.

As the primary model, we examined the **1a** + **2** reaction. Although the overall character of the reaction profiles for the individual competing pathways (**A**–**D**) is broadly similar, the quantitative description of the corresponding critical points varies. For each pathway, two critical points were identified between the energy level of the separated reactants and the final product well: a molecular pre-reaction complex (**MC**) and a transition state (**TS**). In every case, the first stage of the transformation involves the formation of a molecular pre-reaction complex (**MCA** for pathway **A**, **MCB** for pathway **B**, **MCC** for pathway **C**, and **MCD** for pathway **D**). This step is accompanied by a pronounced decrease in the enthalpy of the system (exceeding 10 kcal mol^−1^) and importantly proceeds without any activation barrier (Table 2).

It should be emphasized that already at this early stage of the reaction, the addend substructures adopt a mutual spatial orientation identical to that later observed in the transition state and ultimately retained in the product. Consequently, the identified intermediates should be regarded as orientational complexes. Similar complexes have previously been located using DFT methods in 32CA pathways involving N-nitrile oxides [72,73], azides [74,75], diazo compounds [75,76,77], ylides [78], and other 4-π-electron components [79,80]. Experimental evidence for the formation of pre-reaction complexes has also been reported for the reactions of ozone with ethene [81] and acetylene [82,83], based on microwave spectrometry data [84,85,86].

Although the reacting centers are already aligned in their target positions, the formation of new bonds has not yet commenced. Both the C3–C4 and C5–O1 interatomic distances remain outside the ranges typical for nascent C–C and C–O bonds in transition states [87,88,89]. This indicates that no transfer of electron density should occur between the substructures. Analysis of the GEDT indices confirms this expectation (Table 3).

The gradual approach of the reactive centers leads to the conversion of the pre-reaction complexes into the corresponding transition states (TSA for pathway A, TSB for pathway B, TSC for pathway C, and TSD for pathway D, respectively). Their character was confirmed by vibrational analysis, which in each case revealed a single imaginary frequency. Formation of these TSs is accompanied by an increase in the reaction-system enthalpy by several kcal/mol (Table 2). Notably, these values are relatively low compared with other 32CAs involving nitrones [90,91].

The kinetically most favorable pathway is pathway A. This conclusion is fully consistent with the experimentally observed selectivity: 3,4-*trans*-4,5-*trans*-2-methyl-3-phenyl-4-methylsulfonyl-5-carbomethoxyisoxazolidine **4a** was indeed identified in the reaction mixture (Scheme 1) [34]. In addition, two other products were detected, most likely the 3,4-*cis* and 3,4-*trans* isomers of 2-methyl-3-phenyl-4-carbomethoxy-5-ethylsulfonylisoxazolidines **6a** and **7a** [34]. Pathway B, which would lead to 3,4-*cis*-4,5-*trans*-2-methyl-3-phenyl-4-methylsulfonyl-5-carbomethoxyisoxazolidine **5a**, should be regarded as kinetically inaccessible under the examined competitive conditions. Considering the activation barriers, the reaction pathways can be ordered as A < C < D < B (Table 2).

**Figure 5:**
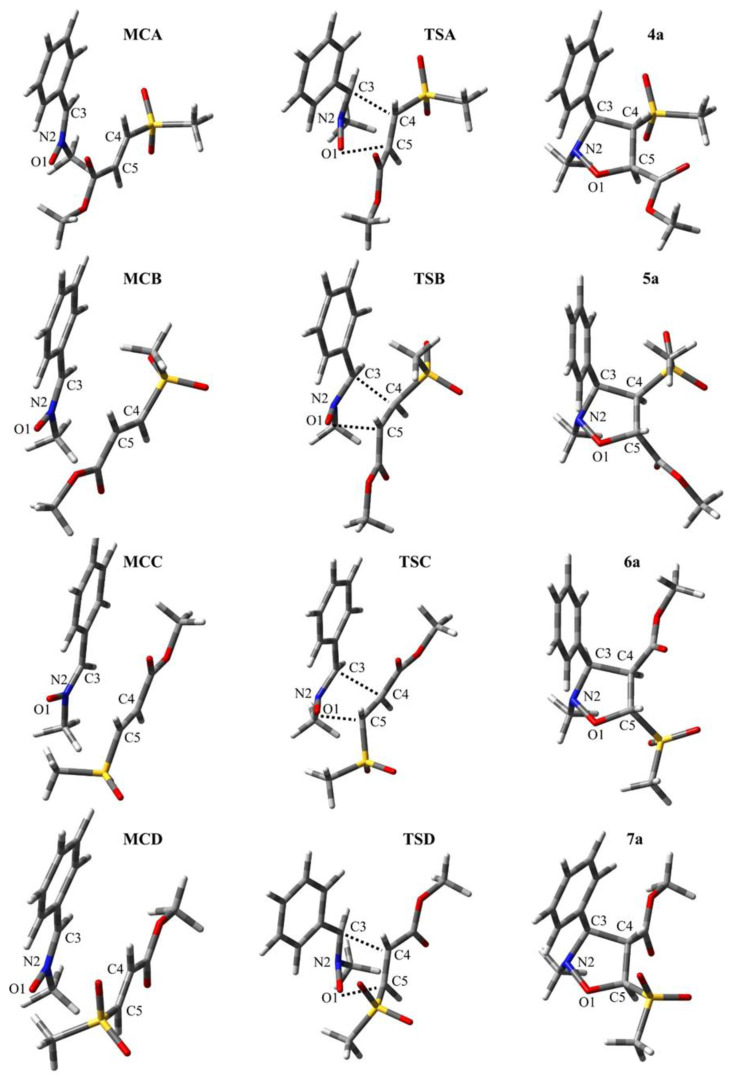
Views of critical structures for 32CA reaction of (*Z*)-C-phenyl-N-methylnitrone (**1a**) with (*E*)-3-(methylsulfonyl)-propenoic acid (**2**) according to ωb97xD/6-311G(d,p) calculations in benzene solution (PCM).

All localized transition states adopt a biplanar geometry characteristic of 32CA reactions involving allylic TACs. In each case, two new bonds essential for forming the isoxazolidine ring, C3–C4 and C5–O1, are established simultaneously (Figure 5). These structures display a relatively high degree of synchronicity, which is expected because both reaction centers on the alkene are electrophilically activated by EWG substituents (Table 3).

Given the nucleophilic character of the nitrone and the electrophilic character of the alkene, a highly polar transition state could be anticipated in this type of reaction, as noted previously for related nitrone 32CAs [92]. However, the transition structures identified here do not display high polarity. The GEDT values for all localized TSs remain below 0.15 e (Table 3), which confirms their low-polar character. This finding clarifies the apparent inconsistency between the predictions based on global and local CDFT reactivity indices and the actual properties of the transition states. Although the CDFT descriptors correctly classify nitrones as nucleophiles and alkenes as electrophiles, the mutual activation of both reacting centers leads to synchronous bond formation with only limited charge separation at the transition state.

IRC analyses show that, for each pathway, the localized TS is the only critical point connecting the pre-reaction complex valley with the product (Figure 5). Attempts to optimize potential acyclic intermediates, whether biradical or zwitterionic, were unsuccessful. These results indicate that the reaction proceeds through a concerted, single-step mechanism characterized by low polarity and a high degree of synchronicity.

Quantum-chemical calculations carried out for the remaining reactions in the series provided analogous results. The conclusions regarding both selectivity and the molecular mechanism are consistent across all systems examined.

### 2.2. Predicted Biological Activity of the Isoxazolidine Derivatives Formed in the Studied 32CAs

Isoxazolidines and their derivatives constitute a versatile class of five-membered heterocycles whose relevance in medicinal chemistry continues to grow due to their diverse biological and pharmacological properties [93,94]. Their saturated N,O-heterocyclic framework combines conformational rigidity with favorable hydrogen-bonding capabilities, which makes isoxazolidines valuable structural motifs in the design of bioactive molecules and enzyme inhibitors, comparable to other privileged scaffolds such as morpholines and piperazines [95,96]. These compounds exhibit a broad spectrum of biological activities, including anticancer, antibacterial, antifungal, antioxidant, and anti-inflammatory effects [97,98]. Certain conjugates have demonstrated potent cytotoxicity against human cancer cell lines with low-micromolar IC_50_ values, while others showed significant antibacterial and antifungal activity in biochemical assays [99,100]. These characteristics make isoxazolidine derivatives promising candidates for further exploration in drug discovery and molecular design.

In view of the diverse applications documented for related isoxazolidine scaffolds, we decided to examine the prospective application potential of the heterocycles characterized in this work. The fundamental physicochemical characteristics, together with selected pharmacokinetic properties, were evaluated using the SwissADME online platform [101]. These results were subsequently analyzed in light of the commonly accepted drug-likeness criteria proposed by Lipinski et al. [102], Ghose et al. [103], Veber et al. [104], Egan et al. [105], as well as Muegge et al. [106]. In addition, the probable biological activities and potential application routes of the investigated isoxazolidine derivatives (**4**–**11**) were predicted through the PASS 2024 software [107].

It should be underline that, because both SwissADME and PASS rely on two-dimensional structural descriptors generated from canonical SMILES, which do not reliably differentiate individual stereoisomers, all computational predictions in this study were performed using a single representative configuration for each isoxazolidine derivative. This approach ensures methodological consistency and prevents the introduction of artifacts resulting from incomplete stereochemical encoding in the input structure. It is also consistent with current cheminformatics practice, as tools operating on SMILES-based fingerprints or similarity metrics typically provide identical or highly similar output for molecules that differ only in stereochemistry. Consequently, the ADME parameters, drug-likeness assessment and predicted biological activity profiles reported in this work correspond to the same defined structural representation for each compound [108,109,110]. These analyses constitute a foundational evaluation intended to deliver initial understanding of the ADME and predicted biological profiles of the investigated isoxazolidine derivatives (**4**–**11**) and to inform subsequent in-depth studies.

#### 2.2.1. Computational Evaluation of ADME and Pharmacokinetic Profiles

**Figure 6:**
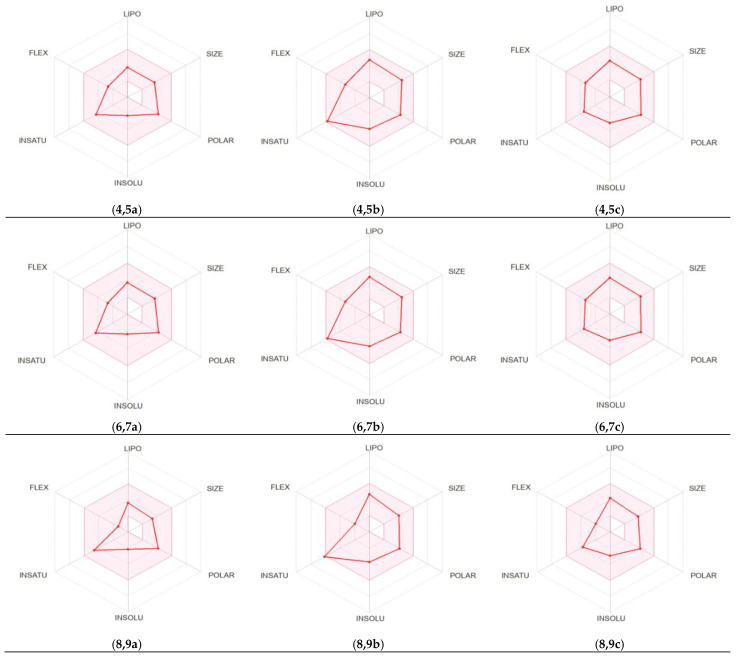

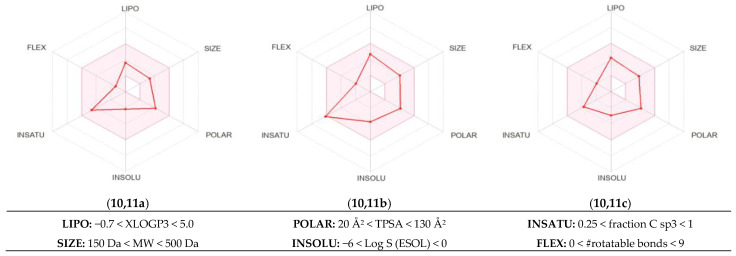
The SwissADME [101] bioavailability radars generated for isoxazolidine (**4**–**11**), illustrate their overall drug-likeness, where the pink region represents the recommended intervals for lipophilicity, molecular size, polarity, aqueous solubility, saturation, and molecular flexibility.

ADME analyses are crucial in the early stages of drug discovery because obtaining experimental pharmacokinetic data is often time-consuming and expensive. As a result, computational methods are increasingly used to predict ADME profiles and improve the efficiency of preliminary screening. In this study the key physicochemical properties of the investigated isoxazolidine derivatives (**4**–**11**), including lipophilicity, solubility, predicted pharmacokinetic behavior and overall medicinal chemistry suitability, were evaluated. The drug-likeness of these molecules was assessed using the SwissADME platform [111], which provides reliable in silico predictions that support the identification and optimization of potential drug candidates [112,113]. The calculated parameters for compounds (**4**–**11**) are summarized in Table 4, whereas their bioavailability radars are shown in Figure 6.

Analysis of the in silico physicochemical parameters and predicted ADME profiles summarized in Table 4 shows that all investigated isoxazolidine derivatives (**4**–**11**) comply with the major drug-likeness criteria applied in this study. In particular, every compound in this series satisfies the accepted limits for molecular weight, number of rotatable bonds, and hydrogen-bond donor/acceptor counts, and displays favorable Topological Polar Surface Area (TPSA) values (Table 4). TPSA, which represents the total surface area of polar atoms (typically nitrogen, oxygen, and sulfur), is a key indicator of membrane permeability and overall drug bioavailability. In general, TPSA values below 140 Å^2^ are associated with good oral bioavailability, whereas values below 90 Å^2^ tend to support blood–brain barrier permeation [114,115]. For all considered compounds (**4**–**11**), the calculated TPSA values are close to 80 Å^2^ (Table 4), which is consistent with good membrane permeability and overall favorable oral bioavailability, according to these computational predictions.

Molar refractivity (MR) is another highly relevant parameter because it describes both the polarizability and the steric volume of a molecule, which together determine how effectively the compound interacts with biological targets. According to the *Ghose* filter, the optimal MR range spans 40–130 cm^3^/mol, which corresponds to molecular dimensions and electronic responsiveness that typically support efficient and selective binding within protein active sites [103]. Compounds with MR values below this threshold are often small and only weakly polarizable, which may limit the strength of their noncovalent interactions and lead to rapid metabolic clearance, ultimately reducing pharmacological potency. In contrast, excessively high MR values may indicate bulky, rigid structures with reduced conformational adaptability. Such molecules frequently suffer from impaired membrane permeability, diminished solubility, and an increased propensity for nonspecific interactions that can negatively impact bioavailability and safety [116,117]. All isoxazolidine derivatives (**4**–**11**) display MR values in the range of 69–96 cm^3^/mol (Table 4), which, according to these in silico estimates, fall well within the optimal interval, confirming that the compounds possess balanced molecular size and polarizability, consistent with favorable interaction potential toward biological targets and promising overall computationally predicted pharmacokinetic behavior.

The evaluation of lipophilicity and aqueous solubility indicates that all isoxazolidines (**4**–**11**) display favorable lipophilic profiles and sufficient water solubility. These properties play a central role in absorption and metabolic processing because lipophilicity facilitates membrane permeation, while aqueous solubility enables adequate compound distribution in physiological fluids. Both features contribute directly to effective gastrointestinal uptake [118,119], and lipophilicity additionally modulates susceptibility to metabolic enzymes, thereby influencing molecular stability and clearance [120,121]. The consensus Log Po/w values [122,123] fall within the optimal range for drug-like molecules and vary depending on the substituent attached to the nitrogen atom of the isoxazolidine ring. In particular, compounds (**4**–**11**) bearing a methyl group show values close to 1.00, compounds with a *tert*-butyl substituent reach approximately 1.70, and analogs containing a phenyl ring exceed 2.00 (Table 4). This trend reflects the increasing hydrophobic character introduced by progressively bulkier substituents and suggests correspondingly enhanced membrane affinity, which may improve cellular uptake while simultaneously requiring careful consideration of metabolic stability and distribution. A similar substituent-dependent trend is observed for the aqueous solubility of the isoxazolidines (**4**–**11**). Their solubility varies substantially and is strongly influenced by the substituent attached to the nitrogen atom of the isoxazolidine ring. Compounds bearing a methyl group exhibit solubility values of approximately 1–2 mg/mL, derivatives containing a tert-butyl substituent reach around 0.3 mg/mL, and analogs with a phenyl group display markedly lower solubility of about 0.01–0.05 mg/mL (Table 4). This decrease in solubility correlates with the increasing hydrophobic surface area introduced by bulkier substituents, which promotes stronger interactions with lipid environments and reduces water compatibility, a factor relevant to absorption, distribution, and overall computationally predicted pharmacokinetic performance [111].

SwissADME predictions indicate that all isoxazolidines (**4**–**11**) display favorable gastrointestinal (GI) absorption (Table 4). High GI absorption suggests that the molecules can effectively traverse the intestinal epithelium and reach systemic circulation, which is crucial for achieving adequate oral bioavailability and therapeutic action [124,125]. The pharmacokinetic parameters summarized in Table 4 also show that each of the tested compounds (**4**–**11**) has low permeability across the blood–brain barrier (BBB). Substances that penetrate the BBB can influence central nervous system function, which is relevant for CNS-targeted therapies. However, limited BBB permeability is advantageous for molecules intended to act peripherally, as it minimizes the likelihood of unintended neurological effects [126]. Finally, the SwissADME predictions show that all isoxazolidines (**4**–**11**) display differentiated inhibitory patterns toward cytochrome P450 isoforms (Table 4).

The extent of CYP inhibition varies depending on the structural features of each compound, indicating that small changes in substituent type or steric environment can significantly influence interactions with metabolic enzymes.

The presented information is fully consistent with the bioavailability radars, and no issues arise apart from a moderate increase in unsaturation for the compounds bearing two phenyl substituents (**4**–**11b**), which still remain within the acceptable range (Figure 6). Moreover, it is worth noting that the predicted SwissADME properties of the resulting regioisomeric products remain essentially unchanged, with only minor differences observed in lipophilicity (Table 4). All of these observations should be understood as in silico predictions, providing guidance for future experimental studies rather than confirmed pharmacological outcomes.

#### 2.2.2. PASS Computational Bioactivity Analysis

Due to the encouraging biological profiles of the isoxazolidine derivatives (**4**–**11**), their potential activity spectrum was explored using the PASS tool [55], accessed via the Way2Drug platform [107]. PASS employs a computational approach that predicts drug-like behavior, possible mechanisms of action, pharmacological effects, toxicity risks, and other features relevant at the early stages of drug development [127]. Although the method relies solely on 2D structural descriptors, it offers fast and reliable estimations that can support both further in silico analyses and future experimental work [128,129]. The output is presented as the probability of activity (Pa) relative to inactivity (Pi), with a compound considered potentially active when Pa exceeds Pi [55]. A Pa value above 0.700 denotes a strong likelihood of activity and makes the compound a good candidate for laboratory validation, whereas values between 0.500 and 0.700 indicate moderate confidence. Predictions below 0.500 are regarded as low-probability outcomes [55]. For all examined derivatives (**4**–**11**), the biological activities that fulfilled the Pa > Pi and Pa > 0.700 criteria are collected in Table 5.

The PASS prediction shows that the main biological potential of the studied isoxazolidines (**4**–**11**) is associated with modulation of the serotonin system, including antagonism of 5-HT2A and 5-HT2C receptors and stimulation of serotonin release, while the predicted CYP2H substrate activity reflects structural features relevant to metabolism but is of limited significance for human pharmacology (Table 5). In general, 5-HT2 antagonists can modulate neurotransmission in the central nervous system, influencing mood, cognition, and cardiovascular as well as metabolic processes [130,131]. Specifically, 5-HT2A antagonism is often associated with effects on perception and psychotropic activity [132], while 5-HT2C antagonism may regulate appetite, energy balance, and serotonergic signaling [133]. Stimulation of serotonin release suggests an additional mechanism to enhance serotonergic neurotransmission, potentially affecting mood and other physiological responses [134]. In contrast, the predicted CYP2H substrate activity indicates that these compounds possess structural features compatible with metabolism by P450 enzymes; however, as CYP2H is an avian isoform, this prediction has limited relevance for human pharmacology [135].

Based on the PASS prediction, two important in silico observations can be drawn. First, the predicted biological activity of the investigated isoxazolidine derivatives (**4**–**11**) is strongly influenced by the EWG present in the ethenyl moiety. In particular, for compounds where the EWG is a methoxycarbonyl group, the main predicted activity of these compounds (**4**–**7**) is predicted as substrates of CYP2H, an avian cytochrome P450 isoform (Table 5). In contrast, when the EWG is a cyano group, the predicted activities of derivatives (**8**–**11**) are primarily related to 5-HT2 antagonism or serotonin release stimulation (Table 5).

It should be emphasized that both the PASS and ADME analyses presented here are purely in silico predictions. They are intended to provide preliminary insights and suggest potential directions for future experimental studies rather than to assert confirmed biological activities. These results complement the mechanistic investigations and highlight possible functional trends, but their pharmacological relevance must be validated through comprehensive laboratory testing.

## 3. Materials and Methods

All computational work was performed with the GAUSSIAN 16 [136] program suite on the Ares cluster hosted by the AGH CYFRONET regional computing center in Cracow, Poland. The DFT computations were conducted with the hybrid ωb97xD functional [137], which combines long-range exchange treatment with an additional semiclassical London-dispersion correction. For all atoms, the 6-311G(d,p) basis set [138,139] was employed, providing d-polarization functions for second-row elements and p-polarization functions for hydrogen. The MEDT approach was employed as the general theoretical tool for examining the mechanisms of reactions involving organic and inorganic systems [140,141,142], with demonstrated applicability to cycloaddition processes [143,144]. All relevant stationary points were optimized in the gas phase at 298 K and 1 atm and subsequently verified by vibrational frequency calculations. The analyses showed that the initial reactants and the products represent true minima, as their Hessian matrices exhibit no imaginary frequencies.

The electronic characteristics of nitrones (**1a**–**c**) and the ethenes (**2** and **3**) were examined using Conceptual Density Functional Theory (CDFT), following the guidelines proposed by Domingo [145,146,147]. The electrophilic Parr functions (Pk^+^) and nucleophilic Parr functions (Pk^−^) were derived from the Atomic Spin Density (ASD) values of the corresponding radical ionic species [148,149].

Optimizations of the transition states (TSs) were carried out using the Berny method [150,151]. Frequency analyses confirmed the nature of the TSs by showing only one imaginary mode. To trace the connection between TSs and minimum-energy structures, intrinsic reaction coordinate (IRC) paths were computed [152] using the second-order González–Schlegel integration procedure [153,154]. Solvent effects of benzene were incorporated through full reoptimization of the gas-phase geometries at the same computational level, employing the polarizable continuum model (PCM) [155] within the self-consistent reaction field (SCRF) formalism [156,157].

The Global Electron Density Transfer (GEDT) [64] values were estimated by the Natural Population Analysis (NPA) [158,159] using the equation GEDT (f) = charge qf were q are the atoms of a framework (f) at the TSs. Indices of single bond development (l) were calculated according to the formula [160,161]:

$$l_{X-Y}=1-\frac{{r\;}_{X-Y}^{\mathrm{TS}}-{r\;}_{X-Y}^{P}}{{r\;}_{X-Y}^{P}}$$

where r^TS^_X−Y_ is the distance between the reaction centers X and Y in the transition structure and r^P^_X−Y_ is the same distance in the corresponding product.

The physicochemical and pharmacokinetic characteristics of the studied compounds were predicted using the SwissADME web platform [101]. This tool allowed for the assessment of absorption, distribution, metabolism, and excretion (ADME) parameters, offering a thorough overview of molecular behavior in a biological context. To evaluate the drug-likeness of the designed molecules, several widely recognized predictive models were applied, including the criteria formulated by Lipinski et al. [102], Ghose et al. [103], Veber et al. [104], Egan et al. [105], and Muegge et al. [106]. Together, these guidelines enabled a systematic analysis of molecular features that affect oral bioavailability and therapeutic potential. Additionally, the PASS online tool [107] was employed to estimate the likelihood of specific biological activities. Based on structure-activity relationship (SAR) modeling, this approach provided complementary insights into the potential pharmacological profiles of the isoxazolidine derivatives under investigation.

GaussView 6.0 software [162] was used to visualize molecular geometries of all the systems, as well as 3D representations of the HOMO and LUMO orbitals, MEP surfaces, and the radical anion and radical cation species. In turn, the bioavailability radar plots were generated using the SwissADME online platform [101].

## 4. Conclusions and Future Perspective

The ωB97X-D/6-311G(d,p) (PCM) quantum-chemical calculations allow for a comprehensive assessment of both the regio- and stereochemistry, as well as the molecular mechanism, of the (3+2) cycloaddition reactions between (*Z*)-C-phenyl-N-alkyl(phenyl)nitrones and (*E*)-3-(methylsulfonyl)-propenoic acid derivatives. The results indicate that the reactions proceed through a single-step mechanism involving highly synchronous formation of the C3–C4 and C5–O1 bonds. Pre-reaction orientational complexes are formed without an activation barrier and already adopt the spatial arrangement characteristic of the final products, emphasizing the role of molecular alignment in determining reaction outcomes. The reaction proceeds through nucleophilic attack of the nitrone oxygen center on the vinyl carbon of (*E*)-3-(methylsulfonyl)-propenoic acid derivatives. Both vinyl carbons of the ethene exhibit similar electrophilic character, which makes the prediction of regioselectivity challenging. Consequently, according to CDFT analysis, the regioselectivity of the process cannot be unambiguously determined, highlighting the subtle interplay of electronic factors in directing the cycloaddition outcome. Despite the expected polar character based on global and local CDFT descriptors, the transition states exhibit low polarity, as confirmed by GEDT values below 0.15 e, highlighting limited charge separation during bond formation.

Among the examined 32CA pathways, route A is predicted to be the kinetically most favorable pathway across all the reactions analyzed. The remaining pathways display higher activation barriers, indicating lower kinetic preference. Experimental results show partial correspondence with the computational predictions. The main limitations of the experimental data are due to the fragmentary nature of the reported results, the lack of consistent yield information, and the incomplete description of the remaining possible products, which prevents a clear assessment of their relative contributions. While the major product is consistent with route A, minor products likely corresponding to pathways C and D are also observed. This indicates that although route A is kinetically favored, contributions from alternative routes cannot be completely excluded, reflecting the subtle electronic and steric factors that complicate the regio- and stereoselectivity of these 32CA reactions. The comparison with the available experimental data underlines the relevance of our computational analysis while also highlighting the limitations inherent to the fragmentary experimental information.

As a future perspective, the analysis of the physicochemical parameters and predicted ADME profiles indicates that all isoxazolidine products formed in the studied reactions exhibit favorable drug-like characteristics. These compounds display properties consistent with good molecular stability, adequate solubility, and suitable permeability, suggesting their potential for effective absorption and distribution in a biological context. What is more, PASS predictions further indicate that the isoxazolidine derivatives exhibit pharmacological potential, particularly in modulation of the serotonin system, including antagonism of 5-HT2A and 5-HT2C receptors and stimulation of serotonin release. The nature of the EWG substituent significantly influences predicted biological activity, with methoxycarbonyl-substituted derivatives showing CYP2H substrate activity, whereas cyano-substituted compounds predominantly display 5-HT2 antagonism or serotonin release stimulation. These findings collectively underscore the interplay between structural features and biological potential, confirming the designed isoxazolidine derivatives as promising candidates for further pharmacological investigation. We hope that the inclusion of these analyses will make our manuscript a useful reference for researchers interested in both the synthetic and potential biological aspects of these compounds.

## Figures and Tables

**Table 1:** ωb97xD/6-311G(d,p) global reactivity indices of nitrones (**1a**–**c**) and (*E*)-3-(methylsulfonyl)-propenoic acid derivatives (**2**–**3**), presented in gas phase in electronvolts [eV].

	1a	1b	1c	2	3
HOMO energy	−7.67	−7.60	−7.64	−10.83	−10.23
LUMO energy	0.34	0.44	−0.25	−0.93	−0.52
Electronic chemical potential, μ	−3.67	−3.58	−3.95	−5.88	−5.38
Chemical hardness, η	8.01	8.05	7.39	9.90	9.72
Global electrophilicity, ω	0.84	0.80	1.05	1.75	1.49
Global nucleophilicity, N	3.73	3.79	3.75	0.57	1.16

**Table 2:** Energetical parameters of 32CA reactions of nitrones (**1a**–**c**) with (*E*)-3-(methylsulfonyl)-propenoic acid derivatives (**2**–**3**) according to ωb97xD/6-311G(d,p) calculations in benzene (PCM) (ΔH, ΔG are in kcal/mol; ΔS are in cal/mol·K).

Reaction	Path	Transition	ΔH	ΔS	ΔG	ΔH	ΔS	ΔG	Transition	Path	Reaction
**1a**+**2**	**A**	**1a**+**2**→**MCA**	−11.9	−40.9	0.3	−11.3	−39.1	0.4	**1a**+**3**→**MCA**	**A**	**1a**+**3**
		**1a**+**2**→**TSA**	3.3	−50.3	18.3	3.8	−48.5	18.3	**1a**+**3**→**TSA**		
		**1a**+**2**→**4a**	−28.4	−48.5	−13.9	−25.6	−49.6	−10.8	**1a**+**3**→**8a**		
	**B**	**1a**+**2**→**MCB**	−12.5	−43.2	0.4	−10.3	−38.2	1.1	**1a**+**3**→**MCB**	**B**	
		**1a**+**2**→**TSB**	8.4	−51.9	23.9	11.2	−50.5	26.2	**1a**+**3**→**TSB**		
		**1a**+**2**→**5a**	−26.3	−53.2	−10.4	−22.9	−53.4	−7.0	**1a**+**3**→**9a**		
	**C**	**1a**+**2**→**MCC**	−13.6	−42.8	−0.8	−10.7	−39.7	1.1	**1a**+**3**→**MCC**	**C**	
		**1a**+**2**→**TSC**	4.4	−52.6	20.0	6.1	−49.4	20.8	**1a**+**3**→**TSC**		
		**1a**+**2**→**6a**	−30.3	−52.7	−14.6	−27.1	−50.7	−11.9	**1a**+**3**→**10a**		
	**D**	**1a**+**2**→**MCD**	−11.5	−38.3	−0.1	−8.9	−41.2	3.3	**1a**+**3**→**MCD**	**D**	
		**1a**+**2**→**TSD**	5.5	−49.0	20.1	6.5	−48.7	21.0	**1a**+**3**→**TSD**		
		**1a**+**2**→**7a**	−28.1	−50.1	−13.2	−26.2	−54.1	−10.1	**1a**+**3**→**11a**		
**1b**+**2**	**A**	**1b**+**2**→**MCA**	−13.1	−32.0	−3.6	−11.6	−33.1	−2.0	**1b**+**3**→**MCA**	**A**	**1b**+**3**
		**1b**+**2**→**TSA**	1.1	−45.2	14.6	1.8	−43.5	21.5	**1b**+**3**→**TSA**		
		**1b**+**2**→**4b**	−32.1	−44.7	−18.8	−28.9	−44.2	−12.4	**1b**+**3**→**8b**		
	**B**	**1b**+**2**→**MCB**	−14.0	−39.7	−2.1	−11.8	−32.8	−2.0	**1b**+**3**→**MCB**	**B**	
		**1b**+**2**→**TSB**	6.6	−47.4	20.7	7.5	−46.9	21.5	**1b**+**3**→**TSB**		
		**1b**+**2**→**5b**	−30.4	−50.7	−15.3	−26.8	−48.1	−12.4	**1b**+**3**→**9b**		
	**C**	**1b**+**2**→**MCC**	−14.7	−35.9	−4.0	−11.6	−34.3	−3.5	**1b**+**3**→**MCC**	**C**	
		**1b**+**2**→**TSC**	3.8	−47.3	17.9	5.8	−45.4	19.3	**1b**+**3**→**TSC**		
		**1b**+**2**→**6b**	−32.3	−45.2	−18.8	−29.5	−45.3	−16.0	**1b**+**3**→**10b**		
	**D**	**1b**+**2**→**MCD**	−12.1	−34.4	−1.8	−11.6	−33.1	−1.7	**1b**+**3**→**MCD**	**D**	
		**1b**+**2**→**TSD**	2.7	−44.8	16.1	4.9	−43.1	17.8	**1b**+**3**→**TSD**		
		**1b**+**2**→**7b**	−32.7	−45.6	−19.2	−27.3	−42.3	−14.7	**1b**+**3**→**11b**		
**1c**+**2**	**A**	**1c**+**2**→**MCA**	−8.1	−42.3	4.5	−11.8	−40.5	0.3	**1c**+**3**→**MCA**	**A**	**1c**+**3**
		**1c**+**2**→**TSA**	4.6	−51.0	19.8	5.2	−50.2	20.2	**1c**+**3**→**TSA**		
		**1c**+**2**→**4c**	−26.3	−49.4	−11.5	−24.0	−49.8	−9.1	**1c**+**3**→**8c**		
	**B**	**1c**+**2**→**MCB**	−12.7	−42.1	−0.1	−8.8	−35.3	1.7	**1c**+**3**→**MCB**	**B**	
		**1c**+**2**→**TSB**	8.3	−54.7	24.6	11.0	−52.4	26.6	**1c**+**3**→**TSB**		
		**1c**+**2**→**5c**	−25.2	−54.3	−9.0	−22.1	−52.2	−6.5	**1c**+**3**→**9c**		
	**C**	**1c**+**2**→**MCC**	−13.3	−42.8	−0.6	−11.9	−40.6	0.2	**1c**+**3**→**MCC**	**C**	
		**1c**+**2**→**TSC**	6.0	−55.5	22.5	7.5	−52.9	23.2	**1c**+**3**→**TSC**		
		**1c**+**2**→**6c**	−28.1	−52.2	−12.5	−25.2	−50.3	−10.2	**1c**+**3**→**10c**		
	**D**	**1c**+**2**→**MCD**	−13.9	−47.4	0.3	−9.7	−33.7	0.4	**1c**+**3**→**MCD**	**D**	
		**1c**+**2**→**TSD**	6.0	−49.9	20.9	6.5	−50.0	21.4	**1c**+**3**→**TSD**		
		**1c**+**2**→**7c**	−29.0	−50.5	−13.9	−24.9	−46.4	−11.0	**1c**+**3**→**11c**		

**Table 3:** Key parameters of critical structures for 32CA reactions of nitrones (**1a**–**c**) with alkenes (**2**–**3**) according to ωb97xD/6-311G(d,p) calculations in benzene solution (PCM).

Reaction	Path	Structure	Interatomic Distances [Å]					l		Δl	GEDT *
			O1–N2	N2–C3	C3–C4	C4–C5	C5–O1	C3–C4	C5–O1		[e]
**1a**+**2**	**A**	**MCA**	1.274	1.303	3.203	1.325	2.788				
		**TSA**	1.288	1.344	2.119	1.392	2.008	0.640	0.591	0.049	0.147
		**4a**	1.419	1.461	1.558	1.530	1.425				
	**B**	**MCB**	1.274	1.303	3.410	1.324	3.019				
		**TSB**	1.279	1.348	2.078	1.390	2.122	0.659	0.517	0.142	0.146
		**5a**	1.432	1.462	1.550	1.526	1.431				
	**C**	**MCC**	1.273	1.303	3.532	1.322	3.332				
		**TSC**	1.306	1.332	2.291	1.393	1.867	0.536	0.673	0.137	0.141
		**6a**	1.437	1.461	1.565	1.520	1.407				
	**D**	**MCD**	1.271	1.304	3.256	1.323	3.108				
		**TSD**	1.299	1.337	2.211	1.392	1.916	0.588	0.646	0.058	0.117
		**7a**	1.425	1.460	1.566	1.524	1.414				
**1b**+**2**	**A**	**MCA**	1.276	1.307	3.173	1.323	3.176				
		**TSA**	1.286	1.348	2.123	1.387	2.052	0.637	0.564	0.073	0.125
		**4b**	1.404	1.464	1.558	1.526	1.429				
	**B**	**MCB**	1.275	1.307	3.431	1.324	3.031				
		**TSB**	1.272	1.356	2.035	1.386	2.244	0.683	0.419	0.264	0.128
		**5b**	1.431	1.469	1.545	1.526	1.419				
	**C**	**MCC**	1.281	1.305	4.293	1.322	4.788				
		**TSC**	1.307	1.335	2.278	1.392	1.892	0.539	0.659	0.120	0.127
		**6b**	1.425	1.461	1.559	1.515	1.411				
	**D**	**MCD**	1.277	1.307	4.025	1.322	3.294				
		**TSD**	1.294	1.344	2.171	1.385	1.996	0.605	0.595	0.010	0.097
		**7b**	1.414	1.462	1.556	1.523	1.420				
**1c**+**2**	**A**	**MCA**	1.274	1.302	3.223	1.324	2.973				
		**TSCA**	1.283	1.347	2.060	1.395	2.027	0.671	0.572	0.099	0.138
		**4c**	1.423	1.467	1.550	1.523	1.420				
	**B**	**MCB**	1.274	1.301	3.730	1.323	2.982				
		**TSB**	1.263	1.355	1.996	1.391	2.245	0.707	0.406	0.301	0.161
		**5c**	1.449	1.466	1.544	1.525	1.409				
	**C**	**MCC**	1.278	1.300	4.070	1.322	4.779				
		**TSC**	1.305	1.331	2.273	1.394	1.856	0.544	0.674	0.130	0.129
		**6c**	1.444	1.460	1.561	1.515	1.400				
	**D**	**MCD**	1.273	1.302	3.329	1.323	3.454				
		**TSD**	1.299	1.336	2.170	1.395	1.900	0.599	0.623	0.024	0.094
		**7c**	1.453	1.477	1.549	1.514	1.379				
**1a**+**3**	**A**	**MCA**	1.274	1.304	3.229	1.329	2.760				
		**TSA**	1.284	1.346	2.097	1.401	2.003	0.658	0.595	0.063	0.195
		**8a**	1.419	1.461	1.562	1.538	1.426				
	**B**	**MCB**	1.283	1.299	5.174	1.329	3.092				
		**TSB**	1.278	1.347	2.110	1.396	2.099	0.646	0.528	0.118	0.199
		**9a**	1.425	1.462	1.559	1.541	1.426				
	**C**	**MCC**	1.275	1.303	3.312	1.329	2.979				
		**TSC**	1.314	1.328	2.359	1.406	1.790	0.494	0.725	0.231	0.201
		**10a**	1.438	1.457	1.566	1.534	1.404				
	**D**	**MCD**	1.277	1.302	4.682	1.325	3.211				
		**TSD**	1.301	1.335	2.222	1.398	1.899	0.586	0.654	0.068	0.049
		**11a**	1.425	1.456	1.571	1.540	1.411				
**1b**+**3**	**A**	**MCA**	1.281	1.305	5.586	1.326	2.975				
		**TSA**	1.281	1.351	2.095	1.395	2.059	0.658	0.559	0.099	0.175
		**8b**	1.405	1.464	1.561	1.534	1.428				
	**B**	**MCB**	1.278	1.303	4.754	1.328	3.063				
		**TSB**	1.270	1.355	2.059	1.392	2.226	0.673	0.448	0.225	0.179
		**9b**	1.416	1.464	1.551	1.534	1.434				
	**C**	**MCC**	1.284	1.305	3.879	1.327	3.150				
		**TSC**	1.314	1.330	2.347	1.404	1.812	0.496	0.713	0.217	0.189
		**10b**	1.432	1.462	1.561	1.526	1.408				
	**D**	**MCD**	1.281	1.305	4.778	1.326	3.457				
		**TSD**	1.301	1.340	2.256	1.396	1.882	0.556	0.659	0.103	0.213
		**11b**	1.413	1.476	1.563	1.528	1.404				
**1c**+**3**	**A**	**MCA**	1.275	1.303	3.241	1.328	2.856				
		**TSCA**	1.278	1.351	2.034	1.403	2.038	0.688	0.560	0.128	0.194
		**8c**	1.427	1.472	1.550	1.532	1.415				
	**B**	**MCB**	1.278	1.301	5.492	1.327	2.898				
		**TSB**	1.268	1.351	2.062	1.396	2.156	0.672	0.485	0.187	0.200
		**9c**	1.428	1.460	1.554	1.535	1.423				
	**C**	**MCC**	1.278	1.299	4.411	1.326	4.861				
		**TSC**	1.316	1.325	2.367	1.407	1.764	0.483	0.736	0.253	0.194
		**10c**	1.449	1.460	1.561	1.526	1.396				
	**D**	**MCD**	1.279	1.300	5.092	1.326	3.339				
		**TSD**	1.303	1.334	2.192	1.402	1.875	0.603	0.661	0.058	0.163
		**11c**	1.429	1.466	1.569	1.535	1.400				

* GEDT(*f*) = charge *q*f.

**Table 4:** Drug-likeness parameters for isoxazolidines (**4**–**11**), calculated using the SwissADME [101].

		4,5a	6,7a	4,5b	6,7b	4,5c	6,7c	8,9a	10,11a	8,9b	10,11b	8,9c	10,11c
*Physchem. properties*	**MW [g/mol]**	299	299	361	361	341	341	266	266	328	328	308	308
	**#heavy atoms**	20	20	25	25	23	23	18	18	23	23	21	21
	**#arom. heavy atoms**	6	6	12	12	6	6	6	6	12	12	6	6
	**#rotatable bonds**	4	4	5	5	5	5	2	2	3	3	3	3
	**#H-bond acceptors**	6	6	5	5	6	6	5	5	4	4	5	5
	**#H-bond donors**	0	0	0	0	0	0	0	0	0	0	0	0
	**Molar refractivity**	76.28	76.28	96.52	96.52	90.74	90.74	69.94	69.94	90.18	90.18	84.40	84.40
	**TPSA [Å^2^]**	81.29	81.29	81.29	81.29	81.29	81.29	78.78	78.78	78.78	78.78	78.78	78.78
*Lipophilicity*	**Log P_o/w_ (iLOGP)**	1.91	2.21	2.20	2.58	2.38	2.62	1.37	1.62	1.75	2.13	1.85	2.04
	**Log P_o/w_ (XLOGP3)**	1.01	1.01	2.79	2.79	1.99	1.99	0.88	0.88	2.66	2.66	1.86	1.86
	**Log P_o/w_ (WLOGP)**	0.94	1.14	2.51	2.72	2.10	2.31	1.29	1.49	2.86	2.97	2.45	2.66
	**Log P_o/w_ (MLOGP)**	0.82	0.82	2.10	2.10	1.59	1.59	0.55	0.55	1.87	1.87	1.34	1.34
	**Log P_o/w_ (SILICOS-IT)**	0.13	0.13	1.22	1.22	0.95	0.95	0.19	0.19	1.28	1.28	0.99	0.99
	**Consensus Log P_o/w_**	0.96	1.06	2.17	2.28	1.80	1.89	0.85	0.94	2.08	2.20	1.70	1.78
*Water solubility*	**Log S (ESOL)**	−2.29	−2.29	−3.86	−3.86	−3.07	−3.07	−2.16	−2.16	−3.74	−3.74	−2.94	−2.94
	**solubility [mg/mL]**	1.53	1.53	0.049	0.049	0.288	0.288	1.84	1.84	0.059	0.059	0.356	0.356
	**Log S (Ali)**	−2.31	−2.31	−4.15	−4.15	−3.32	−3.32	−2.12	−2.12	−3.97	−3.97	−3.14	−3.14
	**solubility [mg/mL]**	1.48	1.48	0.025	0.025	0.162	0.162	2.03	2.03	0.035	0.035	0.226	0.226
	**Log S (SILICOS-IT)**	−2.38	−2.38	−4.47	−4.47	−3.18	−3.18	−2.40	−2.40	−4.50	−4.50	−3.21	−3.21
	**solubility [mg/mL]**	1.25	1.25	0.012	0.012	0.227	0.227	1.06	1.06	0.010	0.010	0.191	0.191
*Pharmacokinetics*	**CYP1A2 inhibitor**	No	No	Yes	Yes	No	No	No	No	No	No	No	No
	**CYP2C19 inhibitor**	No	No	Yes	Yes	No	No	No	No	Yes	Yes	No	No
	**CYP2C9 inhibitor**	No	No	No	No	No	No	No	No	No	No	No	No
	**CYP2D6 inhibitor**	No	No	No	No	No	No	No	No	No	No	No	No
	**CYP3A4 inhibitor**	No	No	Yes	Yes	No	No	No	No	Yes	Yes	No	No
	**IG absorption**	High	High	High	High	High	High	High	High	High	High	High	High
	**BBB permeant**	No	No	No	No	No	No	No	No	No	No	No	No
*Druglikeness*	**Lipinski et al. (Pfizer)** [102]	✓	✓	✓	✓	✓	✓	✓	✓	✓	✓	✓	✓
	**Ghose et al. (Amgen)** [103]	✓	✓	✓	✓	✓	✓	✓	✓	✓	✓	✓	✓
	**Veber et al. (GSK)** [104]	✓	✓	✓	✓	✓	✓	✓	✓	✓	✓	✓	✓
	**Egan et al. (Pharmacia)** [105]	✓	✓	✓	✓	✓	✓	✓	✓	✓	✓	✓	✓
	**Muegge et al. (Bayer)** [106]	✓	✓	✓	✓	✓	✓	✓	✓	✓	✓	✓	✓

**Table 5:** The PASS prediction [107] for isoxazolidine (**4**–**11**) indicates their main potential activities, with results expressed as the probability of activity (Pa) values above 0.700.

	4,5a	6,7a	4,5b	6,7b	4,5c	6,7c	8,9a	10,11a	8,9b	10,11b	8,9c	10,11c
CYP2H substrate	0.788	0.882	0.718	0.853	0.76	0.870	-	-	-	-	-	-
5 Hydroxytryptamine 2C antagonist	-	-	0.759	-	-	-	-	-	0.877	-	-	-
5 Hydroxytryptamine 2A antagonist	-	-	0.724	-	-	-	-	-	0.820	-	-	-
5 Hydroxytryptamine 2 antagonist	-	-	-	-	-	-	-	-	0.733	-	-	-
5 Hydroxytryptamine release stimulant	-	-	-	-	-	-	-	-	-	-	-	0.719

## Data Availability

The original contributions presented in this study are included in the article/Supplementary Materials. Further inquiries can be directed to the corresponding authors.

## Supplementary Materials

The following supporting information can be downloaded at: https://www.mdpi.com/article/10.3390/molecules30244738/s1, Tables S1–S29: Thermochemistry and cartesian coordinates of molecules.
