# Supplementary material for: On the Question of the Regio-, Stereoselectivity and the Molecular Mechanism of the (3+2) Cycloaddition Reaction Between (Z)-C-Phenyl-N-alkyl(phenyl)nitrones and (E)-3-(Methylsulfonyl)-propenoic Acid Derivatives
Source: Molecules. 2025 Dec 11;30(24):4738. doi: 10.3390/molecules30244738 (PMC12736254; doi:10.3390/molecules30244738)
Supplement: Supplementary file 1 [file molecules-30-04738-s001.zip › molecules-4040923-supplementary.pdf]

SUPPLEMENTARY MATERIALS

On the question of the regio- stereoselectivity and the molecular mechanism of the (3+2) cycloaddition reaction between (Z)-C-phenyl-N-alkyl(phenyl)nitrones and (E)-3-(methylsulfonyl)-propenoic acid derivatives

Martyna Ząbkowska [1], Karolina Kula [1,*], Volodymyr Diychuk [2] and Radomir Jasiński [1,*]

[1] Cracow University of Technology, Faculty of Chemical Engineering and Technology, Warszawska 24, 31-155 Cracow, Poland;

[2] Yuriy Fedkovych Chernivtsi National University, Department of Chemistry and Food Examination, Kotsiubynskoho 2, 58-002, Chernivtsi, Ukraine

* Correspondence addresses: karolina.kula@pk.edu.pl (K.K); radomir.jasinski@pk.edu.pl

Table S1. Thermochemistry and cartesian coordinates of molecule (1a) (ωb97xD/6-311G(d,p), in benzene solution (PCM)).

Zero-point correction =	0.156457 (Hartree/Particle)
Thermal correction to Energy =	0.165095 (Hartree/Particle)
Thermal correction to Enthalpy =	0.166039 (Hartree/Particle)
Thermal correction to Gibbs Free Energy =	0.122417 (Hartree/Particle)
Sum of electronic and zero-point Energies =	-439.980370 (Hartree/Particle)
Sum of electronic and thermal Energies =	-439.971733 (Hartree/Particle)
Sum of electronic and thermal Enthalpies =	-439.970789 (Hartree/Particle)
Sum of electronic and thermal Free Energies =	-440.014411 (Hartree/Particle)

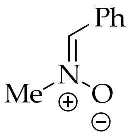

1a

Center	Coordinates (Angstroms) X	Y	Z
C	-1.07811900	-0.72295500	-0.00000400
H	-1.29674100	-1.78167100	-0.00000600
N	-2.12127000	0.06362400	-0.00000300
O	-2.10284400	1.33009200	0.00000000
C	0.31464000	-0.31025500	-0.00000100
C	1.27724300	-1.33321200	0.00000200
C	0.75042300	1.02291900	0.00000000
C	2.62964100	-1.03860100	0.00000400
H	0.95404800	-2.36944100	0.00000100
C	2.11035700	1.30839300	0.00000300
H	0.01396100	1.81197300	-0.00000100
C	3.05236900	0.28796200	0.00000500
H	3.35653500	-1.84277400	0.00000600
H	2.43417400	2.34312000	0.00000300
H	4.11093500	0.52169200	0.00000700
C	-3.47319800	-0.52270000	-0.00000500
H	-3.43364500	-1.61008600	-0.00001100
H	-3.98388300	-0.15411600	0.88827800
H	-3.98388500	-0.15410600	-0.88828300

Table S2. Thermochemistry and cartesian coordinates of molecule (**1b**) (ωb97xD/6-311G(d,p), in benzene solution (PCM)).

Zero-point correction =	0.209462 (Hartree/Particle)
Thermal correction to Energy =	0.220250 (Hartree/Particle)
Thermal correction to Enthalpy =	0.221194 (Hartree/Particle)
Thermal correction to Gibbs Free Energy =	0.171859 (Hartree/Particle)
Sum of electronic and zero-point Energies =	-631.633531 (Hartree/Particle)
Sum of electronic and thermal Energies =	-631.622743 (Hartree/Particle)
Sum of electronic and thermal Enthalpies =	-631.621798 (Hartree/Particle)
Sum of electronic and thermal Free Energies =	-631.671133 (Hartree/Particle)

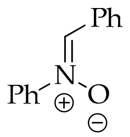

1b

Center	Coordinates (Angstroms) X	Y	Z
C	-0.46455900	0.45286200	0.00009500
H	-0.12638300	1.47594900	0.00029200
N	0.46699600	-0.46615900	-0.00007100
O	0.22978400	-1.71314000	-0.00024300
C	2.33695600	1.22771400	-0.00032500
C	1.88838300	-0.09054600	-0.00004400
C	2.79622700	-1.14033300	0.00026400
C	4.15857500	-0.87337100	0.00031500
C	4.61727000	0.43680700	0.00005200
C	3.70149100	1.48222700	-0.00027100
H	1.65989300	2.07037700	-0.00061500
H	2.41125600	-2.14942200	0.00045700
H	4.86133600	-1.69814200	0.00056200
H	5.68063700	0.64497400	0.00008900
H	4.04651100	2.50934100	-0.00049600
C	-1.89731300	0.22965300	0.00005800
C	-2.70616000	1.37996800	0.00029100
C	-2.52022100	-1.02802700	-0.00019200
C	-4.08604100	1.28182600	0.00027400
H	-2.23995600	2.36019400	0.00048700
C	-3.90684800	-1.11542000	-0.00021000
H	-1.90617000	-1.91470800	-0.00036900
C	-4.69345500	0.02882700	0.00002200
H	-4.69065900	2.18141200	0.00045600
H	-4.37502400	-2.09323600	-0.00040700
H	-5.77450900	-0.05162200	0.00000700

Table S3. Thermochemistry and cartesian coordinates of molecule (1c) (ωb97xD/6-311G(d,p), in benzene solution (PCM)).

Zero-point correction =	0.241390 (Hartree/Particle)
Thermal correction to Energy =	0.253767 (Hartree/Particle)
Thermal correction to Enthalpy =	0.254711 (Hartree/Particle)
Thermal correction to Gibbs Free Energy =	0.202811 (Hartree/Particle)
Sum of electronic and zero-point Energies =	-557.841913 (Hartree/Particle)
Sum of electronic and thermal Energies =	-557.829536 (Hartree/Particle)
Sum of electronic and thermal Enthalpies =	-557.828592 (Hartree/Particle)
Sum of electronic and thermal Free Energies =	-557.880492 (Hartree/Particle)

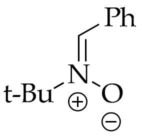

1c

Center	Coordinates (Angstroms) X	Y	Z
C	-0.10062200	0.55344400	-0.00005100
H	-0.37272100	1.59607100	-0.00004400
N	-1.08623700	-0.30135500	-0.00007700
O	-0.94029100	-1.56168100	0.00000700
C	1.31929000	0.23714400	-0.00002200
C	2.20313800	1.32944000	0.00000400
C	1.85634300	-1.05848200	-0.00002400
C	3.57399700	1.13926700	0.00002600
H	1.80335900	2.33872800	0.00000600
C	3.23431500	-1.23962500	-0.00000300
H	1.18287400	-1.90133400	-0.00004500
C	4.09650800	-0.15121800	0.00002200
H	4.23737900	1.99661900	0.00004500
H	3.63552900	-2.24691900	-0.00000600
H	5.16978600	-0.30410000	0.00003900
C	-2.53878400	0.15154400	0.00001100
C	-3.16662800	-0.44272500	1.26141900
C	-2.69412300	1.66908300	0.00063000
C	-3.16646500	-0.44166700	-1.26197500
H	-3.03372300	-1.52343400	1.27378800
H	-2.70494800	-0.02040500	2.15790300
H	-4.23411200	-0.21181700	1.27952800
H	-2.26219500	2.12970400	-0.89111600
H	-3.76243600	1.89531800	0.00058500
H	-2.26244000	2.12896100	0.89287700
H	-4.23393900	-0.21070600	-1.28004000
H	-2.70464500	-0.01862000	-2.15804300
H	-3.03359300	-1.52236700	-1.27522100

Table S4. Thermochemistry and cartesian coordinates of molecule (**2**) (ωb97xD/6-311G(d,p), in benzene solution (PCM)).

Zero-point correction =	0.134695 (Hartree/Particle)
Thermal correction to Energy =	0.145957 (Hartree/Particle)
Thermal correction to Enthalpy =	0.146902 (Hartree/Particle)
Thermal correction to Gibbs Free Energy =	0.096442 (Hartree/Particle)
Sum of electronic and zero-point Energies =	-894.204664 (Hartree/Particle)
Sum of electronic and thermal Energies =	-894.193401 (Hartree/Particle)
Sum of electronic and thermal Enthalpies =	-894.192457 (Hartree/Particle)
Sum of electronic and thermal Free Energies =	-894.242917 (Hartree/Particle)

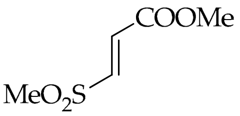

2

Center	Coordinates (Angstroms) X	Y	Z
C	0.24123000	-0.32676900	-0.07326300
H	-0.04514500	-1.36948700	-0.14220200
C	-0.60891200	0.68393300	0.01591400
H	-0.25691000	1.70850700	0.06624000
S	1.99358400	-0.01148300	-0.15725400
O	2.20836300	1.42711300	-0.14316900
O	2.51724200	-0.82078400	-1.24777700
C	2.59302000	-0.68283400	1.38355700
H	2.15510900	-0.11967000	2.20600000
H	3.67618300	-0.56022700	1.36749900
H	2.33283300	-1.73917700	1.43502200
C	-2.08880500	0.51602000	0.04180200
O	-2.84741000	1.44532200	0.12918000
O	-2.46274000	-0.75999500	-0.03768600
C	-3.87504300	-0.99976800	-0.02598200
H	-4.34970800	-0.50373700	-0.87329500
H	-4.31402900	-0.63132700	0.90192900
H	-3.98826300	-2.07790000	-0.10168200

Table S5. Thermochemistry and cartesian coordinates of molecule (**3**) (ωb97xD/6-311G(d,p), in benzene solution (PCM)).

Zero-point correction =	0.089937 (Hartree/Particle)
Thermal correction to Energy =	0.098493 (Hartree/Particle)
Thermal correction to Enthalpy =	0.099437 (Hartree/Particle)
Thermal correction to Gibbs Free Energy =	0.056191 (Hartree/Particle)
Sum of electronic and zero-point Energies =	-758.608705 (Hartree/Particle)
Sum of electronic and thermal Energies =	-758.600150 (Hartree/Particle)
Sum of electronic and thermal Enthalpies =	-758.599205 (Hartree/Particle)
Sum of electronic and thermal Free Energies =	-758.642452 (Hartree/Particle)

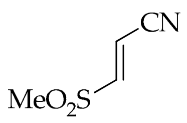

3

**Center**	**Coordinates (Angstroms)**		
	**X**	**Y**	**Z**
C	-0.52939700	0.33444700	0.38536300
H	-0.67621500	1.09339300	1.14477800
C	-1.50001900	-0.28393400	-0.27534600
H	-1.26731500	-1.04819300	-1.01126500
S	1.16448200	-0.14454400	0.08049600
O	1.15622200	-1.20857500	-0.91032600
O	1.78247400	-0.34883300	1.37972300
C	1.86238900	1.31722900	-0.66367700
H	1.34620700	1.51408000	-1.60186700
H	2.91409000	1.09259000	-0.84307600
H	1.76740400	2.14884200	0.03296400
C	-2.87863300	0.01077800	-0.04771700
N	-3.99306900	0.24287600	0.12051600

Table S6. Thermochemistry and cartesian coordinates of molecule (4a) (ωb97xD/6-311G(d,p), in benzene solution (PCM)).

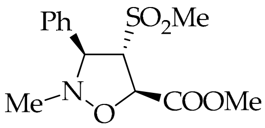

4a

Zero-point correction =	0.296744 (Hartree/Particle)
Thermal correction to Energy =	0.316310 (Hartree/Particle)
Thermal correction to Enthalpy =	0.317255 (Hartree/Particle)
Thermal correction to Gibbs Free Energy =	0.246212 (Hartree/Particle)
Sum of electronic and zero-point Energies =	-1334.228977 (Hartree/Particle)
Sum of electronic and thermal Energies =	-1334.209411 (Hartree/Particle)
Sum of electronic and thermal Enthalpies =	-1334.208467 (Hartree/Particle)
Sum of electronic and thermal Free Energies =	-1334.279509 (Hartree/Particle)

Center	Coordinates (Angstroms)		
	X	Y	Z
C	1.31298800	0.56190800	0.48524500
O	0.52130600	1.23242700	1.46269600
C	-0.79656700	-0.54060400	0.90235800
C	0.38338900	-0.49510100	-0.11467300
H	-0.97081600	-1.55988700	1.25384200
N	-0.35700100	0.27472900	2.03228200
C	0.34719900	-0.47936800	3.06668300
H	-0.38145600	-1.13265300	3.54949000
H	1.17476100	-1.09185200	2.68680300
H	0.71889400	0.22988400	3.80529300
C	-2.07030500	-0.03192600	0.25444700
C	-2.72013000	-0.85008700	-0.66923900
C	-2.58616400	1.22671500	0.53990800
C	-3.87598600	-0.41194200	-1.30104700
H	-2.31444400	-1.83368600	-0.88833200
C	-3.74311100	1.66435400	-0.09639300
H	-2.08296400	1.85364400	1.26512300
C	-4.39006600	0.84910500	-1.01649900
H	-4.37870800	-1.05720900	-2.01257900
H	-4.14080400	2.64701400	0.13148100
H	-5.29324000	1.19224500	-1.50825500
H	2.18644800	0.08863900	0.95410300
H	0.03567900	-0.21508000	-1.10865200
S	1.14076200	-2.12855800	-0.33089300
O	2.04473900	-2.38196000	0.78801300
O	0.05243400	-3.06473700	-0.59052400
C	2.11281300	-1.97247100	-1.81679800
H	2.83141700	-1.16504900	-1.69350100
H	2.61295600	-2.93237700	-1.94315700
H	1.44423900	-1.77005000	-2.65130200
C	1.83327800	1.55277800	-0.53879600
O	2.01738000	1.26169500	-1.69452500
O	2.10257700	2.72741200	-0.00230900
C	2.63001500	3.72144600	-0.89265200
H	2.78303100	4.60524700	-0.27983900
H	3.57278400	3.38145300	-1.32230100
H	1.91742000	3.92600700	-1.69199500

Table S7. Thermochemistry and cartesian coordinates of molecule (4b) (ωb97xD/6-311G(d,p), in benzene solution (PCM)).

Zero-point correction =	0.350054 (Hartree/Particle)
Thermal correction to Energy =	0.372712 (Hartree/Particle)
Thermal correction to Enthalpy =	0.373656 (Hartree/Particle)
Thermal correction to Gibbs Free Energy =	0.295085 (Hartree/Particle)
Sum of electronic and zero-point Energies =	-1525.888995 (Hartree/Particle)
Sum of electronic and thermal Energies =	-1525.866337 (Hartree/Particle)
Sum of electronic and thermal Enthalpies =	-1525.865393 (Hartree/Particle)
Sum of electronic and thermal Free Energies =	-1525.943964 (Hartree/Particle)

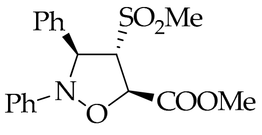

4b

Center	Coordinates (Angstroms) X	Y	Z
C	-0.43306000	1.31196700	-0.34878000
O	-0.47549800	0.45818300	-1.49409600
C	0.49065500	-0.87249200	0.09106700
C	0.51001500	0.59528900	0.61352500
H	0.14382300	-1.54607800	0.87511100
N	-0.49004800	-0.85409000	-0.99526900
C	1.87044400	-1.31464500	-0.35480300
C	2.82934800	-1.58986700	0.61980700
C	2.20222700	-1.43333500	-1.69918200
C	4.10960200	-1.97878500	0.24980000
H	2.56423200	-1.50045200	1.66909900
C	3.48617000	-1.82201300	-2.06675400
H	1.45164000	-1.22923900	-2.45268200
C	4.44171000	-2.09457200	-1.09617500
H	4.84741000	-2.19737900	1.01343500
H	3.73781900	-1.91421600	-3.11730400
H	5.44072300	-2.39997100	-1.38582000
H	-1.43469200	1.39619600	0.09445600
H	1.51656400	1.01224000	0.58938500
S	-0.00903700	0.67185700	2.35488200
O	-1.46548600	0.71485900	2.40386900
O	0.69881300	-0.39521300	3.05413000
C	0.63284100	2.23053500	2.93362400
H	1.71998000	2.19415500	2.89179500
H	0.24689800	3.03211100	2.30745300
H	0.28770100	2.32734800	3.96268900
C	0.02462600	2.69619000	-0.76016200
O	0.75696600	3.37766600	-0.08799400
O	-0.51739900	3.06597400	-1.90527800
C	-0.17012200	4.37701500	-2.37571300
H	-0.69803900	4.49444200	-3.31772700
H	-0.49008800	5.13166600	-1.65673600
H	0.90719400	4.44849200	-2.52642200
C	-1.80355200	-1.32497900	-0.72189100
C	-1.96549100	-2.55588000	-0.08256500
C	-2.92544000	-0.62456300	-1.16177500
C	-3.23922000	-3.05372500	0.14708400
H	-1.10139000	-3.13690700	0.21735000
C	-4.19467900	-1.13788600	-0.92719800
H	-2.79933900	0.30987200	-1.69171200
C	-4.36264200	-2.34771800	-0.26756100
H	-3.35025500	-4.00756100	0.64989000
H	-5.05960300	-0.57958900	-1.26725400
H	-5.35539900	-2.74117500	-0.08537100

Table S8. Thermochemistry and cartesian coordinates of molecule (4c) (ωb97xD/6-311G(d,p), in benzene solution (PCM)).

Zero-point correction =	0.381288 (Hartree/Particle)
Thermal correction to Energy =	0.404806 (Hartree/Particle)
Thermal correction to Enthalpy =	0.405751 (Hartree/Particle)
Thermal correction to Gibbs Free Energy =	0.326882 (Hartree/Particle)
Sum of electronic and zero-point Energies =	-1452.087386 (Hartree/Particle)
Sum of electronic and thermal Energies =	-1452.063867 (Hartree/Particle)
Sum of electronic and thermal Enthalpies =	-1452.062922 (Hartree/Particle)
Sum of electronic and thermal Free Energies =	-1452.141791 (Hartree/Particle)

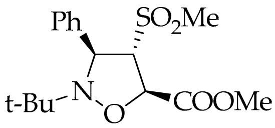

4c

Center	Coordinates (Angstroms) X	Y	Z
C	-1.48964700	0.18969200	0.21073100
O	-0.88850500	1.36904200	0.72374000
C	0.85548600	0.27564300	-0.35056500
C	-0.31258200	-0.71736500	-0.12429000
H	1.13100800	0.27271000	-1.40641600
N	0.33397400	1.59252800	0.03085300
C	2.07815600	-0.11913200	0.45919000
C	2.94941700	-1.08502000	-0.04082500
C	2.32071900	0.44222000	1.70918400
C	4.05114000	-1.48666800	0.70512000
H	2.75765700	-1.52198800	-1.01509900
C	3.42146900	0.03685800	2.45454600
H	1.64993900	1.20720300	2.08116400
C	4.28922200	-0.92798900	1.95561700
H	4.72766400	-2.23368900	0.30523000
H	3.60487600	0.48093600	3.42666800
H	5.15032800	-1.23897300	2.53632500
H	-2.07996300	0.39366800	-0.69002400
H	-0.09070200	-1.40066900	0.69565500
S	-0.61051000	-1.78707700	-1.57678900
O	-1.71146600	-1.22253300	-2.35213900
O	0.66919900	-2.01252800	-2.24142700
C	-1.14663000	-3.32771900	-0.86119600
H	-0.31065600	-3.75996600	-0.31345900
H	-1.98938900	-3.12781900	-0.20182300
H	-1.43228400	-3.96346100	-1.69862100
C	-2.43177200	-0.38669000	1.25106900
O	-2.51301100	-1.56396700	1.50030200
O	-3.17559800	0.55350100	1.80399800
C	-4.12414100	0.11357400	2.78635700
H	-4.63030100	1.01407100	3.12256200
H	-4.83336200	-0.58392500	2.33971500
H	-3.60900600	-0.37064300	3.61634300
C	0.18313800	2.60998700	-1.03931100
C	-0.30686900	3.89012900	-0.35887500
C	1.59046900	2.85097300	-1.59841000
C	-0.78114300	2.22127500	-2.16894200
H	-1.28222500	3.73291600	0.10323800
H	0.39767200	4.20066100	0.41616200
H	-0.39530000	4.69216500	-1.09616400
H	1.95296800	2.01429500	-2.20058700
H	1.57505400	3.73327800	-2.24204400
H	2.29836100	3.02431100	-0.78469100
H	-0.73453900	2.97171800	-2.96218600
H	-0.53738800	1.25487400	-2.61756500
H	-1.81360200	2.18712300	-1.81392900

Table S9. Thermochemistry and cartesian coordinates of molecule (5a) (ωb97xD/6-311G(d,p), in benzene solution (PCM)).

Zero-point correction =	0.297346 (Hartree/Particle)
Thermal correction to Energy =	0.316655 (Hartree/Particle)
Thermal correction to Enthalpy =	0.317599 (Hartree/Particle)
Thermal correction to Gibbs Free Energy =	0.248780 (Hartree/Particle)
Sum of electronic and zero-point Energies =	-1334.225385 (Hartree/Particle)
Sum of electronic and thermal Energies =	-1334.206077 (Hartree/Particle)
Sum of electronic and thermal Enthalpies =	-1334.205133 (Hartree/Particle)
Sum of electronic and thermal Free Energies =	-1334.273951 (Hartree/Particle)

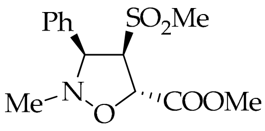

5a

Center	Coordinates (Angstroms) X	Y	Z
C	1.20421700	-0.47161600	0.46515000
O	0.83710500	-1.85429500	0.49144800
C	-0.66409000	-0.93705400	-0.98219800
C	0.43580000	0.12092700	-0.71322800
H	0.91804700	-0.04391700	1.42793400
H	-0.89657800	-0.95354600	-2.04724700
H	1.09750200	0.17379600	-1.58271600
N	0.02214100	-2.18216700	-0.63927000
S	0.02235300	1.86929600	-0.53634900
O	1.30353000	2.53056800	-0.31198900
O	-0.78867800	2.23492800	-1.68904900
C	-0.94964200	2.10870800	0.94343700
H	-1.93568400	1.66799800	0.82491300
H	-0.42228000	1.69816500	1.80205000
H	-1.01848700	3.19294200	1.03792400
C	0.87904100	-2.70143400	-1.70244100
H	0.22989300	-3.02337500	-2.51872400
H	1.62023100	-1.98267700	-2.07080900
H	1.40266000	-3.57371800	-1.31320100
C	-1.94568800	-0.74688500	-0.20155800
C	-3.00702300	-0.07138200	-0.80090300
C	-2.07682900	-1.19448400	1.11254400
C	-4.17358200	0.18158300	-0.08903600
H	-2.91093900	0.28170900	-1.82159500
C	-3.24370300	-0.94200900	1.82249700
H	-1.26431000	-1.74236500	1.57411900
C	-4.29151600	-0.24727100	1.22774800
H	-4.98979300	0.71244400	-0.56509600
H	-3.33601900	-1.29234100	2.84421700
H	-5.19992900	-0.04993700	1.78522100
C	2.71437600	-0.33818900	0.28727200
O	3.29944800	-0.60724400	-0.72810400
O	3.28616500	0.09093500	1.40291400
C	4.70821400	0.27027400	1.34951800
H	4.99480600	0.62178000	2.33687300
H	5.19847400	-0.67594200	1.11893800
H	4.96174600	1.00927400	0.58910900

Table S10. Thermochemistry and cartesian coordinates of molecule (**5b**) (ωb97xD/6-311G(d,p), in benzene solution (PCM)).

Zero-point correction =	0.350539 (Hartree/Particle)
Thermal correction to Energy =	0.372891 (Hartree/Particle)
Thermal correction to Enthalpy =	0.373835 (Hartree/Particle)
Thermal correction to Gibbs Free Energy =	0.298117 (Hartree/Particle)
Sum of electronic and zero-point Energies =	-1525.885961 (Hartree/Particle)
Sum of electronic and thermal Energies =	-1525.863609 (Hartree/Particle)
Sum of electronic and thermal Enthalpies =	-1525.862665 (Hartree/Particle)
Sum of electronic and thermal Free Energies =	-1525.938383 (Hartree/Particle)

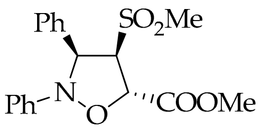

5b

Center	Coordinates (Angstroms) X	Y	Z
C	0.02270700	1.01904900	-0.89267900
O	-0.86544500	0.13802200	-1.56292500
C	0.00788300	-1.06859800	0.21933200
C	0.11699800	0.44356400	0.51772300
H	0.99263700	0.99232200	-1.39763200
H	-0.36252300	-1.58853100	1.10155400
H	-0.76456400	0.74591700	1.09353100
N	-1.05096800	-1.05749200	-0.79828700
S	1.45551700	1.06521800	1.56021400
O	1.09823000	2.44001700	1.88423700
O	1.64095000	0.10240000	2.63626600
C	2.92849500	1.11441500	0.55482500
H	3.72152200	1.42371100	1.23593300
H	3.13669000	0.12591700	0.14892800
H	2.78856600	1.86184800	-0.22451000
C	1.28007100	-1.72757600	-0.26680700
C	2.12995800	-2.32944700	0.66027700
C	1.63430000	-1.72516000	-1.61551400
C	3.33322400	-2.88997400	0.25051500
H	1.85982400	-2.33511500	1.70969800
C	2.83826400	-2.28635400	-2.02329400
H	0.96000300	-1.29625800	-2.34750800
C	3.69388400	-2.86287300	-1.09164500
H	3.98692300	-3.35142400	0.98152300
H	3.10442500	-2.27873400	-3.07401900
H	4.63248600	-3.30016900	-1.41211400
C	-0.50006700	2.44715800	-0.91316200
O	-1.64631300	2.76832300	-0.77034400
O	0.51883500	3.28451000	-1.07516600
C	0.21828800	4.67901300	-0.92094600
H	1.15359100	5.20056000	-1.10397800
H	-0.54162500	4.98249900	-1.64092600
H	-0.13321500	4.86702400	0.09366100
C	-2.38224900	-1.14478600	-0.28587700
C	-2.75033400	-2.36099200	0.29372800
C	-3.30504600	-0.10800700	-0.37772500
C	-4.02683200	-2.52385000	0.80823400
H	-2.03753000	-3.17784800	0.32266900
C	-4.58802900	-0.29226700	0.12863200
H	-3.01996100	0.83058700	-0.83171600
C	-4.95456400	-1.48965400	0.72686100
H	-4.30240100	-3.46970200	1.26047500
H	-5.30265600	0.51987000	0.05707600
H	-5.95545000	-1.62165300	1.12050900

Table S11. Thermochemistry and cartesian coordinates of molecule (**5c**) (ωb97xD/6-311G(d,p), in benzene solution (PCM)).

Zero-point correction =	0.381740 (Hartree/Particle)
Thermal correction to Energy =	0.405008 (Hartree/Particle)
Thermal correction to Enthalpy =	0.405952 (Hartree/Particle)
Thermal correction to Gibbs Free Energy =	0.329387 (Hartree/Particle)
Sum of electronic and zero-point Energies =	-1452.085451 (Hartree/Particle)
Sum of electronic and thermal Energies =	-1452.062182 (Hartree/Particle)
Sum of electronic and thermal Enthalpies =	-1452.061238 (Hartree/Particle)
Sum of electronic and thermal Free Energies =	-1452.137804 (Hartree/Particle)

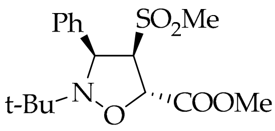

5c

Center	Coordinates (Angstroms) X	Y	Z
C	1.05592800	-0.12915300	-0.76653900
O	0.89231200	1.22321700	-1.12482400
C	-0.73528200	0.79433400	0.49207900
C	0.40707000	-0.23774000	0.60932400
H	0.55286400	-0.76807100	-1.49950200
H	-0.96845200	1.17758300	1.48449500
H	1.11853700	0.09757400	1.36746900
N	-0.13616700	1.85440200	-0.32349900
S	0.07022500	-1.91058900	1.20368100
O	1.38086500	-2.51984500	1.39735700
O	-0.83427200	-1.78927400	2.33860800
C	-0.74336700	-2.81659800	-0.10118200
H	-1.67452900	-2.32764100	-0.37904800
H	-0.05784300	-2.90730500	-0.94200500
H	-0.93813800	-3.79941500	0.32881400
C	-2.00251800	0.26089800	-0.14562200
C	-3.02165300	-0.24655300	0.65737000
C	-2.15003400	0.23697000	-1.53185600
C	-4.15786900	-0.80370300	0.08273300
H	-2.91192000	-0.23237600	1.73544100
C	-3.28381700	-0.32402100	-2.10567900
H	-1.37898100	0.67315300	-2.15650000
C	-4.28741200	-0.85213900	-1.30036000
H	-4.94242700	-1.19900700	0.71753800
H	-3.38745200	-0.34179000	-3.18461800
H	-5.17237600	-1.28847700	-1.74920100
C	2.53731000	-0.47981100	-0.73049600
O	3.38280000	0.18822100	-0.20279000
O	2.74549300	-1.64281000	-1.33663400
C	4.07233000	-2.17630200	-1.22868000
H	4.05476900	-3.11485300	-1.77581800
H	4.79355800	-1.48657400	-1.66744900
H	4.31462500	-2.34794600	-0.17966800
C	0.44924500	3.02426200	0.38459500
C	-0.73457100	3.78581300	0.99038800
C	1.47868100	2.67103100	1.46478300
C	1.10635800	3.90124900	-0.68500200
H	-1.48626400	3.99216500	0.22552700
H	-1.21264500	3.23780300	1.80643500
H	-0.38102900	4.73488800	1.39958800
H	2.29757300	2.08310600	1.04449500
H	1.90014900	3.58925200	1.88117000
H	1.02718200	2.12119700	2.29675100
H	1.43877200	4.83943200	-0.23403600
H	1.97036800	3.40080000	-1.12205800
H	0.39323900	4.12782200	-1.48108700

Table S12. Thermochemistry and cartesian coordinates of molecule (**6a**) (ωb97xD/6-311G(d,p), in benzene solution (PCM)).

Zero-point correction =	0.296793 (Hartree/Particle)
Thermal correction to Energy =	0.316215 (Hartree/Particle)
Thermal correction to Enthalpy =	0.317159 (Hartree/Particle)
Thermal correction to Gibbs Free Energy =	0.248117 (Hartree/Particle)
Sum of electronic and zero-point Energies =	-1334.231967 (Hartree/Particle)
Sum of electronic and thermal Energies =	-1334.212544 (Hartree/Particle)
Sum of electronic and thermal Enthalpies =	-1334.211600 (Hartree/Particle)
Sum of electronic and thermal Free Energies =	-1334.280643 (Hartree/Particle)

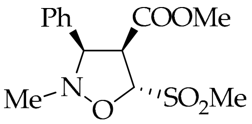

6a

Center	Coordinates (Angstroms) X	Y	Z
C	-1.19388100	-0.22678400	-0.49023800
O	-1.07993700	-1.61292000	-0.27892600
C	0.54913600	-0.71154600	1.06089300
C	-0.32924800	0.47608400	0.54343000
H	-0.89851700	0.01998300	-1.51263600
H	0.75427800	-0.57509400	2.12326400
H	-0.92862800	0.85638600	1.37349300
N	-0.31571600	-1.87877100	0.90864300
C	-1.23114500	-2.08174500	2.02825400
H	-1.90749100	-2.89544100	1.76846300
H	-0.62883000	-2.39619700	2.88245300
H	-1.82353800	-1.19726800	2.28628700
C	1.86185300	-0.84947500	0.31817400
C	2.98765400	-0.19042800	0.80899600
C	1.96540700	-1.58496800	-0.86030200
C	4.19634100	-0.25138900	0.12654500
H	2.91726900	0.37665800	1.73163800
C	3.17452600	-1.64803000	-1.54061200
H	1.09843900	-2.11263300	-1.23867300
C	4.29116900	-0.97878500	-1.05333700
H	5.06529200	0.26252900	0.52175600
H	3.24443300	-2.22324400	-2.45680500
H	5.23280500	-1.02925800	-1.58788800
S	-2.94267200	0.31859300	-0.34151000
O	-3.40456500	0.07783800	1.02156000
O	-3.00436000	1.66971200	-0.88558900
C	-3.80038500	-0.80633500	-1.42576900
H	-3.42728400	-0.66900200	-2.43949300
H	-3.62986900	-1.82051200	-1.06990000
H	-4.85585300	-0.54265600	-1.36536100
C	0.47899900	1.61472000	-0.03679100
O	0.63221600	1.83207200	-1.20755800
O	1.03193900	2.32744200	0.94316700
C	1.92408300	3.36844100	0.52634900
H	1.40135300	4.08467800	-0.10770700
H	2.26537300	3.84453500	1.44187100
H	2.76412700	2.93873200	-0.02187500

Table S13. Thermochemistry and cartesian coordinates of molecule (**6b**) (ωb97xD/6-311G(d,p), in benzene solution (PCM)).

Zero-point correction =	0.349454 (Hartree/Particle)
Thermal correction to Energy =	0.372260 (Hartree/Particle)
Thermal correction to Enthalpy =	0.373204 (Hartree/Particle)
Thermal correction to Gibbs Free Energy =	0.294872 (Hartree/Particle)
Sum of electronic and zero-point Energies =	-1525.889487 (Hartree/Particle)
Sum of electronic and thermal Energies =	-1525.866681 (Hartree/Particle)
Sum of electronic and thermal Enthalpies =	-1525.865737 (Hartree/Particle)
Sum of electronic and thermal Free Energies =	-1525.944069 (Hartree/Particle)

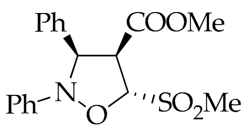

6b

Center	Coordinates (Angstroms) X	Y	Z
C	-0.23170400	1.19781700	-0.66428800
O	-0.88275400	0.14728700	-1.34480600
C	0.45550200	-0.88069000	0.24109400
C	0.32701400	0.62585500	0.62262500
H	0.54935800	1.63431900	-1.29216600
H	0.30562800	-1.48754800	1.13302700
H	-0.39918900	0.71083400	1.43501100
N	-0.67922700	-1.08432200	-0.65714800
C	1.78279700	-1.23421100	-0.39452500
C	2.83967700	-1.62929700	0.42358000
C	1.98234500	-1.14269800	-1.76952800
C	4.08514000	-1.91328400	-0.12216100
H	2.68694300	-1.71518800	1.49473500
C	3.22742200	-1.42879300	-2.31467500
H	1.15791600	-0.85822600	-2.41282700
C	4.28225300	-1.80981700	-1.49374300
H	4.89867500	-2.22371900	0.52352700
H	3.37337800	-1.35534500	-3.38625400
H	5.25263300	-2.03208800	-1.92240200
S	-1.41823200	2.55533000	-0.32368300
O	-2.46337900	2.05234800	0.55768300
O	-0.62752300	3.70778300	0.08382600
C	-2.13302200	2.86121000	-1.92860500
H	-2.88076000	3.64044000	-1.78367300
H	-1.34880400	3.20314500	-2.60231200
H	-2.59394100	1.94195200	-2.28648700
C	1.63856700	1.25430200	1.03663100
O	2.32351300	1.95013900	0.33880400
O	1.94907900	0.88660800	2.27827900
C	3.22515400	1.33168400	2.75587600
H	3.27207200	2.42075900	2.75078600
H	3.30680800	0.94908400	3.76982100
H	4.01918500	0.92876900	2.12510200
C	-1.87246200	-1.61309400	-0.09362100
C	-1.78273300	-2.87203500	0.50867100
C	-3.10762000	-0.98148900	-0.18793100
C	-2.91329900	-3.46678400	1.04602800
H	-0.83128200	-3.39153600	0.53391600
C	-4.23559500	-1.59561900	0.34422900
H	-3.18478700	-0.00792600	-0.64532200
C	-4.14887200	-2.83243100	0.96739500
H	-2.82849800	-4.44038000	1.51516300
H	-5.19081400	-1.08783800	0.27482100
H	-5.03310500	-3.30162400	1.38188700

Table S14. Thermochemistry and cartesian coordinates of molecule (**6c**) (ωb97xD/6-311G(d,p), in benzene solution (PCM)).

Zero-point correction =	0.380999 (Hartree/Particle)
Thermal correction to Energy =	0.404496 (Hartree/Particle)
Thermal correction to Enthalpy =	0.405440 (Hartree/Particle)
Thermal correction to Gibbs Free Energy =	0.327898 (Hartree/Particle)
Sum of electronic and zero-point Energies =	-1452.090215 (Hartree/Particle)
Sum of electronic and thermal Energies =	-1452.066718 (Hartree/Particle)
Sum of electronic and thermal Enthalpies =	-1452.065774 (Hartree/Particle)
Sum of electronic and thermal Free Energies =	-1452.143316 (Hartree/Particle)

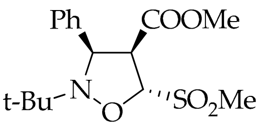

6c

Center	Coordinates (Angstroms) X	Y	Z
C	-0.92120400	-0.58384600	0.67037000
O	-1.17543200	0.75810600	0.97684700
C	0.58279300	0.79044400	-0.52953700
C	-0.08902900	-0.61755700	-0.59503700
H	-0.41151400	-1.09039800	1.49591000
H	0.75029000	1.15048200	-1.54406400
H	-0.71926600	-0.66517500	-1.48471600
N	-0.40545500	1.63855600	0.13003700
C	1.90738400	0.77829500	0.20755900
C	3.08292000	0.54723200	-0.50376900
C	1.97068100	0.95604700	1.58710000
C	4.30432500	0.47693300	0.15501400
H	3.04192800	0.42007200	-1.58095400
C	3.19112900	0.88474400	2.24589000
H	1.06100400	1.16261700	2.13870500
C	4.35994200	0.64133800	1.53355000
H	5.21287900	0.30172000	-0.40998800
H	3.23016600	1.02265300	3.32046300
H	5.31123700	0.58726300	2.05033000
S	-2.51417700	-1.46991000	0.47081900
O	-3.29821700	-0.81711700	-0.57098300
O	-2.19951800	-2.88687200	0.34591100
C	-3.31590100	-1.17023200	2.03509900
H	-2.72101600	-1.62156500	2.82766000
H	-3.41249400	-0.09404000	2.16874800
H	-4.29496500	-1.64426700	1.97169500
C	0.90413000	-1.75710000	-0.62599700
O	1.21404000	-2.43542300	0.31444000
O	1.42279500	-1.87716500	-1.84792600
C	2.46128800	-2.85454000	-1.98656400
H	2.08613400	-3.84505300	-1.72860100
H	2.75966600	-2.81785900	-3.03107000
H	3.29974200	-2.60078200	-1.33580900
C	-1.28223700	2.47109900	-0.73926700
C	-0.39166700	3.63839700	-1.18250400
C	-1.87051100	1.75896300	-1.96422700
C	-2.41660100	3.00878900	0.13458700
H	0.00732200	4.16463200	-0.31328800
H	0.44988000	3.30290600	-1.79497200
H	-0.97788500	4.33777200	-1.78338800
H	-2.48852400	0.90947200	-1.67258100
H	-2.50187800	2.46071300	-2.51526100
H	-1.09388400	1.41916300	-2.65637800
H	-2.96671400	3.77352900	-0.41864300
H	-3.11157400	2.21300400	0.40394900
H	-2.01952600	3.45551600	1.04899000

Table S15. Thermochemistry and cartesian coordinates of molecule (**7a**) (ωb97xD/6-311G(d,p), in benzene solution (PCM)).

Zero-point correction =	0.296493 (Hartree/Particle)
Thermal correction to Energy =	0.316107 (Hartree/Particle)
Thermal correction to Enthalpy =	0.317051 (Hartree/Particle)
Thermal correction to Gibbs Free Energy =	0.246752 (Hartree/Particle)
Sum of electronic and zero-point Energies =	-1334.228614 (Hartree/Particle)
Sum of electronic and thermal Energies =	-1334.209000 (Hartree/Particle)
Sum of electronic and thermal Enthalpies =	-1334.208056 (Hartree/Particle)
Sum of electronic and thermal Free Energies =	-1334.278355 (Hartree/Particle)

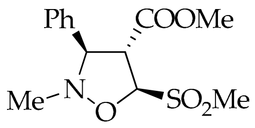

7a

Center	Coordinates (Angstroms) X	Y	Z
C	1.23753600	0.77856100	0.39165500
O	0.46708300	1.12254400	1.52680400
C	-0.41959500	-0.88337300	0.90222200
C	0.69054800	-0.53799300	-0.14758100
H	2.30824100	0.72637000	0.60739800
H	-0.34679600	-1.93269400	1.19648200
H	0.22803800	-0.42180800	-1.12866200
N	-0.10533400	-0.06469200	2.06895000
C	-1.81247500	-0.63465100	0.35737900
C	-2.62708800	0.37515900	0.85200400
C	-2.27924500	-1.44942200	-0.67312400
C	-3.89690300	0.56949200	0.31978700
H	-2.26205700	1.00506100	1.65280500
C	-3.54724100	-1.25672400	-1.20247300
H	-1.64191900	-2.23668400	-1.06592900
C	-4.36063000	-0.24337200	-0.70599200
H	-4.52400200	1.36448300	0.70727100
H	-3.90083500	-1.89659200	-2.00298000
H	-5.35036200	-0.08805000	-1.12006600
S	1.02186300	2.11464300	-0.83410000
O	2.05872000	1.90814000	-1.83810200
O	-0.37385900	2.16250100	-1.23948000
C	1.39001800	3.58184200	0.10822500
H	0.67562300	3.64850900	0.92630700
H	2.41482600	3.51926100	0.47119500
H	1.27620000	4.41929800	-0.57955200
C	0.87077800	-0.67248800	2.96990700
H	1.09588400	0.04290300	3.75990500
H	0.40402200	-1.54945600	3.42123800
H	1.80370400	-0.97079700	2.47415700
C	1.75190700	-1.61248400	-0.26149900
O	2.87321300	-1.54760100	0.17054900
O	1.25814500	-2.67182800	-0.89900300
C	2.14819900	-3.78419400	-1.06087700
H	2.46300900	-4.16191700	-0.08741300
H	1.57794100	-4.53732000	-1.59778700
H	3.02474600	-3.48419300	-1.63536000

Table S16. Thermochemistry and cartesian coordinates of molecule (**7b**) (ωb97xD/6-311G(d,p), in benzene solution (PCM)).

Zero-point correction =	0.349582 (Hartree/Particle)
Thermal correction to Energy =	0.372303 (Hartree/Particle)
Thermal correction to Enthalpy =	0.373247 (Hartree/Particle)
Thermal correction to Gibbs Free Energy =	0.295108 (Hartree/Particle)
Sum of electronic and zero-point Energies =	-1525.890097 (Hartree/Particle)
Sum of electronic and thermal Energies =	-1525.867377 (Hartree/Particle)
Sum of electronic and thermal Enthalpies =	-1525.866433 (Hartree/Particle)
Sum of electronic and thermal Free Energies =	-1525.944572 (Hartree/Particle)

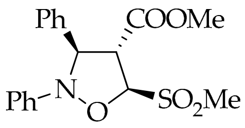

7b

Center	Coordinates (Angstroms) X	Y	Z
C	0.16003400	1.42836900	-0.04808600
O	0.03217900	0.82254000	-1.32620000
C	0.10143700	-0.96205700	0.08704000
C	0.11276300	0.30972400	0.98395500
H	-0.61263000	2.18039900	0.13043000
H	-0.56606000	-1.70316400	0.52295200
H	0.98631200	0.30705100	1.63930800
N	-0.48756600	-0.48221700	-1.16231100
C	1.46694600	-1.58949500	-0.10012900
C	2.07038700	-1.68425200	-1.34703700
C	2.11673200	-2.11145100	1.01677600
C	3.31734600	-2.28555000	-1.47396500
H	1.56365700	-1.28148100	-2.21473700
C	3.36081500	-2.71182400	0.89014900
H	1.64611400	-2.04821400	1.99386700
C	3.96637800	-2.79833900	-0.35888200
H	3.78498600	-2.34774500	-2.45007600
H	3.85824900	-3.11203400	1.76619900
H	4.94064300	-3.26265000	-0.46023600
S	1.75394100	2.32051700	-0.00640600
O	1.67349200	3.21253600	1.14338800
O	2.84695000	1.36740600	-0.10284700
C	1.70153300	3.27884500	-1.50852400
H	1.64320700	2.59155700	-2.35018000
H	0.84198500	3.94650100	-1.47196100
H	2.62953800	3.84939400	-1.53425200
C	-1.11918900	0.42740200	1.87012700
O	-1.85706300	1.37414600	1.91408300
O	-1.25833500	-0.66744600	2.61450000
C	-2.39801000	-0.69445400	3.48431200
H	-3.31560700	-0.62250100	2.89943700
H	-2.34625100	-1.64847000	4.00200200
H	-2.35122500	0.13178800	4.19400100
C	-1.91418600	-0.49304000	-1.21840800
C	-2.57007400	-1.71218900	-1.02955500
C	-2.65501200	0.64862500	-1.51018000
C	-3.95387700	-1.77131600	-1.08911300
H	-1.99705200	-2.61597600	-0.85774600
C	-4.04144100	0.57401200	-1.57383200
H	-2.15143300	1.58758400	-1.69134000
C	-4.69981800	-0.62785300	-1.35581900
H	-4.45037100	-2.72293500	-0.93755100
H	-4.60764800	1.47182500	-1.79341000
H	-5.78088700	-0.67807700	-1.40441900

Table S17. Thermochemistry and cartesian coordinates of molecule (7c) (ωb97xD/6-311G(d,p), in benzene solution (PCM)).

Zero-point correction =	0.380783 (Hartree/Particle)
Thermal correction to Energy =	0.404394 (Hartree/Particle)
Thermal correction to Enthalpy =	0.405339 (Hartree/Particle)
Thermal correction to Gibbs Free Energy =	0.326967 (Hartree/Particle)
Sum of electronic and zero-point Energies =	-1452.091803 (Hartree/Particle)
Sum of electronic and thermal Energies =	-1452.068192 (Hartree/Particle)
Sum of electronic and thermal Enthalpies =	-1452.067248 (Hartree/Particle)
Sum of electronic and thermal Free Energies =	-1452.145620 (Hartree/Particle)

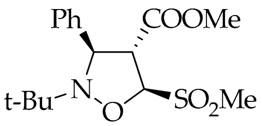

7c

Center	Coordinates (Angstroms) X	Y	Z
C	1.41475900	0.96398800	-0.19998700
O	1.36066200	0.63934500	1.13954000
C	-0.25280200	-0.74216600	0.10568900
C	0.67050300	-0.10940700	-0.96510400
H	2.46231200	1.04587500	-0.49813600
H	-0.09038800	-1.82244900	0.09736900
H	0.09527700	0.30806000	-1.79304300
N	0.18204800	-0.17128300	1.39655500
C	-1.73405400	-0.52189500	-0.16297700
C	-2.58545800	0.04479600	0.77878400
C	-2.26082500	-0.94608900	-1.38386400
C	-3.93937600	0.20198300	0.49996700
H	-2.17371500	0.37696200	1.72310400
C	-3.61104000	-0.78928100	-1.66261300
H	-1.60721700	-1.40404100	-2.11993400
C	-4.45596700	-0.21165400	-0.72041300
H	-4.59018600	0.65180900	1.24130200
H	-4.00472700	-1.11803500	-2.61757000
H	-5.51044600	-0.08675800	-0.93783900
S	0.79850200	2.68319500	-0.51359600
O	1.78451200	3.57703200	0.08015700
O	0.50304300	2.78164500	-1.94130200
C	-0.71469600	2.84418000	0.41289900
H	-1.49483600	2.25940400	-0.06917000
H	-0.53206400	2.50039000	1.42951000
H	-0.95617600	3.90682600	0.39629100
C	1.63769300	-1.12523500	-1.55227800
O	2.83542200	-1.07761200	-1.47497600
O	0.96596000	-2.09434100	-2.17362600
C	1.75774300	-3.13489300	-2.76289300
H	2.35043100	-3.63655400	-1.99721600
H	1.04646500	-3.82399900	-3.21003400
H	2.42060200	-2.71975200	-3.52231400
C	0.60429600	-1.16179300	2.42523100
C	1.02905700	-0.36902800	3.66440300
C	-0.62406800	-2.00689700	2.77325800
C	1.75943900	-2.05497400	1.95060400
H	1.92814700	0.21600800	3.47035200
H	0.23008700	0.31193700	3.96785600
H	1.23323500	-1.05577100	4.48950100
H	-0.99760500	-2.57889700	1.92029600
H	-0.35148600	-2.71980500	3.55448700
H	-1.43671700	-1.38220900	3.14886200
H	2.09478900	-2.68896700	2.77443300
H	1.46154000	-2.71958000	1.13421500
H	2.60428400	-1.45096000	1.61674700

Table S18. Thermochemistry and cartesian coordinates of molecule (**8a**) (ωb97xD/6-311G(d,p), in benzene solution (PCM)).

Zero-point correction =	0.252063 (Hartree/Particle)
Thermal correction to Energy =	0.268805 (Hartree/Particle)
Thermal correction to Enthalpy =	0.269749 (Hartree/Particle)
Thermal correction to Gibbs Free Energy =	0.206446 (Hartree/Particle)
Sum of electronic and zero-point Energies =	-1198.628453 (Hartree/Particle)
Sum of electronic and thermal Energies =	-1198.611711 (Hartree/Particle)
Sum of electronic and thermal Enthalpies =	-1198.610767 (Hartree/Particle)
Sum of electronic and thermal Free Energies =	-1198.674070 (Hartree/Particle)

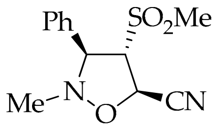

8a

**Center**	Coordinates (Angstroms) **X**	**Y**	**Z**
C	-1.26984500	1.37700100	0.39363900
O	-0.30730200	2.22870200	-0.22408500
C	0.39110800	0.15287100	-0.86973400
C	-0.67027800	-0.03323700	0.26103500
H	0.19336500	-0.52862500	-1.70063800
N	0.20756500	1.52352300	-1.34256300
C	-0.71117500	1.64693700	-2.47245700
H	-0.21780300	1.20745200	-3.34060300
H	-1.67532200	1.14456900	-2.32341600
H	-0.86723800	2.70765900	-2.66411800
C	1.78508800	-0.12175600	-0.33826700
C	2.15335400	-1.44261600	-0.08461500
C	2.68457500	0.90595500	-0.08115000
C	3.41274700	-1.73136600	0.42310600
H	1.44797200	-2.24342000	-0.28700400
C	3.94472700	0.61341500	0.42960800
H	2.39771600	1.92891500	-0.28958300
C	4.31179200	-0.70215200	0.68288900
H	3.69396200	-2.76115900	0.61177800
H	4.64216600	1.41927300	0.62826000
H	5.29574400	-0.92635600	1.07868300
H	-2.23661600	1.43997800	-0.11957200
C	-1.45316600	1.80365200	1.77877900
N	-1.62789500	2.09960300	2.87477200
H	-0.19386600	-0.37183300	1.18074800
S	-1.89147700	-1.30262500	-0.16056900
O	-2.79549300	-0.75062700	-1.16310100
O	-1.14948900	-2.52024600	-0.45364500
C	-2.79830300	-1.53757400	1.35750600
H	-2.11071300	-1.87996500	2.12922900
H	-3.28365900	-0.60350300	1.63909100
H	-3.54525400	-2.30228300	1.14540800

Table S19. Thermochemistry and cartesian coordinates of molecule (**8b**) (ωb97xD/6-311G(d,p), in benzene solution (PCM)).

Zero-point correction =	0.305216 (Hartree/Particle)
Thermal correction to Energy =	0.325110 (Hartree/Particle)
Thermal correction to Enthalpy =	0.326054 (Hartree/Particle)
Thermal correction to Gibbs Free Energy =	0.254497 (Hartree/Particle)
Sum of electronic and zero-point Energies =	-1390.287942 (Hartree/Particle)
Sum of electronic and thermal Energies =	-1390.268047 (Hartree/Particle)
Sum of electronic and thermal Enthalpies =	-1390.267103 (Hartree/Particle)
Sum of electronic and thermal Free Energies =	-1390.338661 (Hartree/Particle)

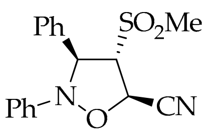

8b

Center	Coordinates (Angstroms) X	Y	Z
C	0.46575200	1.11602600	1.37538100
O	0.47082800	-0.22673700	1.86275300
C	-0.48393700	-0.43795600	-0.20599700
C	-0.51369400	1.07017700	0.19510100
H	-0.11102900	-0.54436900	-1.22583600
N	0.48422200	-1.03611900	0.71483500
C	-1.86321000	-1.06135200	-0.11554000
C	-2.81595700	-0.71781200	-1.07426800
C	-2.19578100	-1.94935000	0.90030600
C	-4.09258600	-1.25948100	-1.01304700
H	-2.55241100	-0.02443600	-1.86726400
C	-3.47628600	-2.48898600	0.95905800
H	-1.44975000	-2.22169000	1.63645900
C	-4.42617900	-2.14626200	0.00553200
H	-4.82650300	-0.99229300	-1.76469400
H	-3.72933300	-3.18166600	1.75360500
H	-5.42261500	-2.57039300	0.05284500
H	1.46843300	1.39716600	1.02981400
C	0.05843100	2.00921700	2.45488700
N	-0.26171400	2.75043600	3.27168700
H	-1.52132400	1.38012200	0.47172800
S	-0.01074700	2.12299100	-1.19266200
O	1.42284200	1.95640500	-1.38442800
O	-0.92613000	1.83824200	-2.28816900
C	-0.32787700	3.77686700	-0.60434000
H	0.30274900	3.98362500	0.25974800
H	-0.06864000	4.44151000	-1.42839900
H	-1.38492300	3.86809800	-0.35969900
C	1.79855700	-1.30035300	0.23525100
C	1.95223400	-1.96494900	-0.98280100
C	2.92167900	-0.99263700	0.99989700
C	3.22231900	-2.27325600	-1.44586200
H	1.08541400	-2.26377600	-1.56021300
C	4.18763200	-1.30998400	0.52330700
H	2.80439300	-0.52050900	1.96582300
C	4.34870700	-1.94251300	-0.70145200
H	3.32782300	-2.78389000	-2.39600900
H	5.05436900	-1.05815900	1.12366800
H	5.33860600	-2.18507500	-1.06836100

Table S20. Thermochemistry and cartesian coordinates of molecule (**8c**) (ωb97xD/6-311G(d,p), in benzene solution (PCM)).

Zero-point correction =	0.336448 (Hartree/Particle)
Thermal correction to Energy =	0.357221 (Hartree/Particle)
Thermal correction to Enthalpy =	0.358165 (Hartree/Particle)
Thermal correction to Gibbs Free Energy =	0.286688 (Hartree/Particle)
Sum of electronic and zero-point Energies =	-1316.487687 (Hartree/Particle)
Sum of electronic and thermal Energies =	-1316.466914 (Hartree/Particle)
Sum of electronic and thermal Enthalpies =	-1316.465969 (Hartree/Particle)
Sum of electronic and thermal Free Energies =	-1316.537446 (Hartree/Particle)

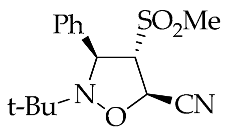

8c

Center	Coordinates (Angstroms) X	Y	Z
C	-1.66978400	0.37787600	0.94737900
O	-0.81843400	1.45192900	1.29833300
C	0.40838400	0.19816300	-0.24689800
C	-0.71327300	-0.67340600	0.37450500
H	0.27586100	0.21954600	-1.33189200
N	0.20503000	1.54575900	0.30833500
C	1.79179900	-0.33892400	0.06761200
C	2.41284200	-1.22100400	-0.81100400
C	2.43692800	0.02720000	1.24623000
C	3.66923900	-1.73584500	-0.51253600
H	1.90625800	-1.51684800	-1.72262800
C	3.68988200	-0.49131200	1.54500300
H	1.95826400	0.73419600	1.91363200
C	4.30955000	-1.37350500	0.66594700
H	4.14780100	-2.41931200	-1.20455400
H	4.18744800	-0.20000600	2.46314000
H	5.29042700	-1.77296700	0.89741300
H	-2.41015700	0.66964800	0.19421500
C	-2.37546700	-0.06304000	2.15092500
N	-2.94915700	-0.44565400	3.06955000
H	-0.31445600	-1.33216400	1.14678900
S	-1.48764700	-1.73605900	-0.87644300
O	-2.29138800	-0.88012600	-1.74178200
O	-0.44238000	-2.56136000	-1.46067100
C	-2.57837900	-2.78642600	0.06533000
H	-1.99279000	-3.33146200	0.80411100
H	-3.35426400	-2.18364500	0.53517700
H	-3.01755500	-3.47592300	-0.65559300
C	-0.06628800	2.64958400	-0.64787900
C	-0.09962200	3.93974100	0.17431400
C	1.13682700	2.69478100	-1.59727000
C	-1.37280300	2.49955500	-1.43912200
H	-0.90791200	3.91290800	0.90633100
H	0.84516200	4.07508900	0.70544300
H	-0.25857300	4.79614500	-0.48577200
H	1.15083900	1.85835900	-2.30024600
H	1.09251300	3.61395700	-2.18511600
H	2.07097300	2.68738600	-1.03114500
H	-1.43461700	3.28844500	-2.19268800
H	-1.44396000	1.53925600	-1.95641600
H	-2.24141100	2.60944700	-0.78455100

Table S21. Thermochemistry and cartesian coordinates of molecule (**9a**) (ωb97xD/6-311G(d,p), in benzene solution (PCM)).

Zero-point correction =	0.252738 (Hartree/Particle)
Thermal correction to Energy =	0.269201 (Hartree/Particle)
Thermal correction to Enthalpy =	0.270145 (Hartree/Particle)
Thermal correction to Gibbs Free Energy =	0.208664 (Hartree/Particle)
Sum of electronic and zero-point Energies =	-1198.623961 (Hartree/Particle)
Sum of electronic and thermal Energies =	-1198.607498 (Hartree/Particle)
Sum of electronic and thermal Enthalpies =	-1198.606554 (Hartree/Particle)
Sum of electronic and thermal Free Energies =	-1198.668035 (Hartree/Particle)

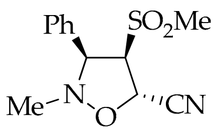

9a

Center	Coordinates (Angstroms) X	Y	Z
C	-1.74283300	-0.35425400	-0.78531500
O	-1.28754000	-1.70261500	-0.86975900
C	-0.05703600	-0.90085000	0.87428800
C	-1.02015200	0.24437100	0.43760000
H	-1.47159200	0.13186700	-1.72449000
H	-0.03279900	-0.94078500	1.96377600
H	-1.73349600	0.43955000	1.24142300
N	-0.74246100	-2.09687500	0.38603800
C	-3.20737800	-0.29223500	-0.66238900
N	-4.35256100	-0.26744800	-0.57234000
S	-0.41266400	1.93395600	0.17198800
O	-1.57799100	2.66550500	-0.30746400
O	0.24560200	2.34005400	1.40379100
C	0.78469600	1.93501600	-1.15483500
H	1.73317100	1.53588000	-0.80715400
H	0.40185600	1.37409800	-2.00586200
H	0.87703300	2.98834500	-1.42138700
C	-1.81283500	-2.57146400	1.25878800
H	-1.34740300	-2.97813700	2.15755000
H	-2.53932500	-1.79958900	1.54790200
H	-2.33699600	-3.37500900	0.74403800
C	1.36389700	-0.80154700	0.36230300
C	2.31359900	-0.14340000	1.14129800
C	1.73930800	-1.33002600	-0.87148500
C	3.61459000	0.01587200	0.68001400
H	2.02867400	0.26991900	2.10217900
C	3.04095100	-1.17244700	-1.33001000
H	1.01151100	-1.86200800	-1.47094900
C	3.97932300	-0.49264600	-0.56109200
H	4.34237800	0.53500400	1.29255600
H	3.32398400	-1.58499900	-2.29161900
H	4.99341700	-0.36905300	-0.92319200

Table S22. Thermochemistry and cartesian coordinates of molecule **(9b)** (ωb97xD/6-311G(d,p), in benzene solution (PCM)).

Zero-point correction =	0.305721 (Hartree/Particle)
Thermal correction to Energy =	0.325403 (Hartree/Particle)
Thermal correction to Enthalpy =	0.326347 (Hartree/Particle)
Thermal correction to Gibbs Free Energy =	0.256631 (Hartree/Particle)
Sum of electronic and zero-point Energies =	-1390.284259 (Hartree/Particle)
Sum of electronic and thermal Energies =	-1390.264577 (Hartree/Particle)
Sum of electronic and thermal Enthalpies =	-1390.263633 (Hartree/Particle)
Sum of electronic and thermal Free Energies =	-1390.333349 (Hartree/Particle)

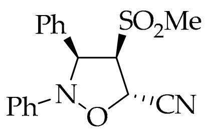

9b

Center	Coordinates (Angstroms) X	Y	Z
C	0.28417800	1.24345900	1.16906700
O	0.81452300	0.02045100	1.69857800
C	-0.10141000	-0.63264900	-0.29470400
C	-0.09112800	0.91829400	-0.28240100
H	-0.58302800	1.49766600	1.78379500
H	0.20423600	-0.96903400	-1.28326400
H	0.70034700	1.25910700	-0.95583700
N	0.97074900	-0.92607900	0.65754500
C	1.25623900	2.33588300	1.29265000
N	2.01729600	3.18667000	1.42104600
S	-1.50223400	1.84604100	-0.94152200
O	-1.13072100	3.24608400	-0.79869700
O	-1.77442800	1.29100400	-2.25809100
C	-2.92450700	1.57144400	0.10389000
H	-3.26121000	0.54137400	0.02691700
H	-2.69091200	1.84877300	1.13010500
H	-3.67531800	2.25436200	-0.29535500
C	-1.41150000	-1.29431300	0.06820900
C	-2.27694500	-1.66307600	-0.96032500
C	-1.78927900	-1.51439400	1.39208400
C	-3.51853200	-2.21528300	-0.67095000
H	-1.99005300	-1.49079200	-1.99155500
C	-3.03009500	-2.06925400	1.67907500
H	-1.11083400	-1.25704900	2.19608800
C	-3.90059600	-2.41299800	0.65074700
H	-4.18502200	-2.49257600	-1.47914300
H	-3.31608100	-2.23686200	2.71100900
H	-4.86895500	-2.84316300	0.87855700
C	2.30014700	-0.95301900	0.13674800
C	2.57193100	-1.82408000	-0.92148900
C	3.33212000	-0.20710400	0.69875700
C	3.85558100	-1.90534900	-1.43861300
H	1.78837700	-2.45555800	-1.32398800
C	4.61604900	-0.30423400	0.17411100
H	3.14029900	0.43548200	1.54589900
C	4.88505000	-1.14176400	-0.89875400
H	4.05225900	-2.58083200	-2.26309000
H	5.40918400	0.28819500	0.61515500
H	5.88734100	-1.20955800	-1.30402100

Table S23. Thermochemistry and cartesian coordinates of molecule (**9c**) (ωb97xD/6-311G(d,p), in benzene solution (PCM)).

Zero-point correction =	0.336591 (Hartree/Particle)
Thermal correction to Energy =	0.357234 (Hartree/Particle)
Thermal correction to Enthalpy =	0.358178 (Hartree/Particle)
Thermal correction to Gibbs Free Energy =	0.287843 (Hartree/Particle)
Sum of electronic and zero-point Energies =	-1316.484581 (Hartree/Particle)
Sum of electronic and thermal Energies =	-1316.463938 (Hartree/Particle)
Sum of electronic and thermal Enthalpies =	-1316.462994 (Hartree/Particle)
Sum of electronic and thermal Free Energies =	-1316.533328 (Hartree/Particle)

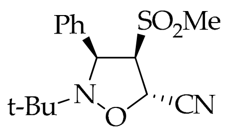

9c

Center	Coordinates (Angstroms) X	Y	Z
C	-0.99148200	1.00682600	-1.06720700
O	-1.39598200	-0.32477400	-1.36475200
C	-0.12732200	-0.59075000	0.52893500
C	-0.31862700	0.93542100	0.31083200
H	-0.29912900	1.31408800	-1.85556900
H	-0.26825200	-0.80650900	1.58736400
H	-0.99220600	1.32372900	1.07671000
N	-1.22232800	-1.18049800	-0.23487100
C	-2.14111100	1.92081700	-1.11106000
N	-3.05854600	2.61011700	-1.17085600
S	1.06506200	2.08868400	0.53132600
O	0.50369500	3.39467400	0.21204400
O	1.60668500	1.83324600	1.85737900
C	2.33361000	1.75717300	-0.68253100
H	2.84956100	0.83011800	-0.44976700
H	1.90081800	1.74062800	-1.68112900
H	3.00673600	2.61047500	-0.59128200
C	1.21566900	-1.14388300	0.09855400
C	2.24177500	-1.24523200	1.03488800
C	1.45156200	-1.52879700	-1.22025700
C	3.50001300	-1.69582300	0.65299600
H	2.06442200	-0.94541900	2.06160500
C	2.70930200	-1.97784800	-1.60095100
H	0.64609200	-1.47922900	-1.94307500
C	3.73836000	-2.05573700	-0.66812800
H	4.29219400	-1.76677000	1.38914000
H	2.88567400	-2.27281900	-2.62902600
H	4.71918500	-2.40635200	-0.96792900
C	-2.50318000	-1.46923000	0.46815900
C	-2.21049000	-2.69144300	1.34657900
C	-3.06238000	-0.32896700	1.33087100
C	-3.52497800	-1.85491200	-0.60290700
H	-1.80622300	-3.50487700	0.74149600
H	-1.49713600	-2.46791500	2.14433200
H	-3.13517600	-3.02881300	1.82007600
H	-3.29069300	0.56026700	0.73980200
H	-3.99792800	-0.65484000	1.79138900
H	-2.38583000	-0.05736200	2.14660600
H	-4.43126200	-2.22785900	-0.12047700
H	-3.79210000	-0.99870700	-1.22311400
H	-3.12312600	-2.63897100	-1.24829300

Table S24. Thermochemistry and cartesian coordinates of molecule (**10a**) (ωb97xD/6-311G(d,p), in benzene solution (PCM)).

Zero-point correction =	0.252134 (Hartree/Particle)
Thermal correction to Energy =	0.268879 (Hartree/Particle)
Thermal correction to Enthalpy =	0.269823 (Hartree/Particle)
Thermal correction to Gibbs Free Energy =	0.207042 (Hartree/Particle)
Sum of electronic and zero-point Energies =	-1198.630799 (Hartree/Particle)
Sum of electronic and thermal Energies =	-1198.614053 (Hartree/Particle)
Sum of electronic and thermal Enthalpies =	-1198.613109 (Hartree/Particle)
Sum of electronic and thermal Free Energies =	-1198.675891 (Hartree/Particle)

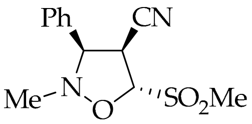

10a

Center	Coordinates (Angstroms) X	Y	Z
C	0.97372300	0.09442100	0.44790600
O	0.72078100	-1.28534500	0.51684600
C	-0.76870800	-0.53645300	-1.05014100
C	0.26744200	0.61271100	-0.81100700
H	0.63855600	0.58197300	1.36625100
H	-0.97090200	-0.62320500	-2.11868900
H	0.97358600	0.62655100	-1.64699000
N	-0.03718000	-1.72238400	-0.62389100
C	0.87863100	-2.26223400	-1.62490200
H	1.43160400	-3.08171300	-1.16728200
H	0.26780200	-2.67008900	-2.43190700
H	1.59271200	-1.53222400	-2.02173100
C	-2.07267500	-0.34605600	-0.30376600
C	-3.06225700	0.45509700	-0.87055500
C	-2.30471200	-0.94297300	0.93298400
C	-4.26330700	0.66757800	-0.20781000
H	-2.89387300	0.92092900	-1.83632800
C	-3.50798700	-0.73076000	1.59488200
H	-1.54806700	-1.58213300	1.37024200
C	-4.48798200	0.07547900	1.02917100
H	-5.02366500	1.29386500	-0.65944800
H	-3.68055300	-1.20147200	2.55603300
H	-5.42543300	0.23878200	1.54819200
C	-0.28552900	1.94897400	-0.67267800
N	-0.72069800	3.00863200	-0.58418100
S	2.78617000	0.41127200	0.33802800
O	3.25346800	-0.08532500	-0.95146100
O	2.98707700	1.80952600	0.68950000
C	3.45149400	-0.63469000	1.61763900
H	3.04415400	-0.31792800	2.57662500
H	3.18998100	-1.66598100	1.39097900
H	4.53112100	-0.48884100	1.59268900

Table S25. Thermochemistry and cartesian coordinates of molecule (**10b**) (ωb97xD/6-311G(d,p), in benzene solution (PCM)).

Zero-point correction =	0.304988 (Hartree/Particle)
Thermal correction to Energy =	0.324954 (Hartree/Particle)
Thermal correction to Enthalpy =	0.325899 (Hartree/Particle)
Thermal correction to Gibbs Free Energy =	0.254839 (Hartree/Particle)
Sum of electronic and zero-point Energies =	-1390.288909 (Hartree/Particle)
Sum of electronic and thermal Energies =	-1390.268942 (Hartree/Particle)
Sum of electronic and thermal Enthalpies =	-1390.267998 (Hartree/Particle)
Sum of electronic and thermal Free Energies =	-1390.339058 (Hartree/Particle)

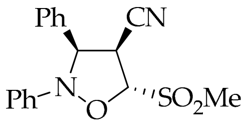

10b

Center	Coordinates (Angstroms) X	Y	Z
C	-0.01160800	1.30212000	-0.30378900
O	-0.50132500	0.30114000	-1.16373500
C	0.71989200	-0.79838800	0.46499100
C	0.29075100	0.59770400	1.01511800
H	0.86771600	1.79103900	-0.73379900
H	0.54741600	-1.55689100	1.22812400
H	-0.64192700	0.47611700	1.57715800
N	-0.24824000	-0.99575200	-0.61282900
C	2.15484400	-0.85579300	-0.01142800
C	3.16896200	-1.07525200	0.91911000
C	2.48363100	-0.68616400	-1.35407100
C	4.49656600	-1.11205700	0.51552700
H	2.92216900	-1.21510100	1.96660000
C	3.81322700	-0.72342000	-1.75653600
H	1.69638000	-0.54080400	-2.08422700
C	4.82151500	-0.93358100	-0.82412000
H	5.27671300	-1.28247900	1.24800400
H	4.06021700	-0.59248400	-2.80380200
H	5.85772100	-0.96320700	-1.14056000
C	1.26275500	1.26865100	1.85960300
N	2.02995300	1.77745900	2.54684200
S	-1.27879900	2.61763900	-0.10669700
O	-2.40245600	2.05736000	0.63164600
O	-0.57886400	3.78454600	0.40503600
C	-1.79674800	2.91754100	-1.78525600
H	-0.93952300	3.26491900	-2.36011200
H	-2.20739500	1.99654900	-2.19484300
H	-2.55908000	3.69429500	-1.72757200
C	-1.45191500	-1.67699400	-0.25668000
C	-1.32079000	-3.03370100	0.04870200
C	-2.70600900	-1.07851700	-0.24324500
C	-2.43764800	-3.77434900	0.40109000
H	-0.34470700	-3.50275900	-0.00844700
C	-3.82127100	-1.83842700	0.09535100
H	-2.81489100	-0.02882900	-0.47018600
C	-3.69609700	-3.18057800	0.42366100
H	-2.32557000	-4.82522000	0.64169000
H	-4.79540600	-1.36342100	0.11010500
H	-4.57021300	-3.76302400	0.68918900

Table S26. Thermochemistry and cartesian coordinates of molecule (**10c**) (ωb97xD/6-311G(d,p), in benzene solution (PCM)).

Zero-point correction =	0.336407 (Hartree/Particle)
Thermal correction to Energy =	0.357177 (Hartree/Particle)
Thermal correction to Enthalpy =	0.358121 (Hartree/Particle)
Thermal correction to Gibbs Free Energy =	0.286881 (Hartree/Particle)
Sum of electronic and zero-point Energies =	-1316.489695 (Hartree/Particle)
Sum of electronic and thermal Energies =	-1316.468924 (Hartree/Particle)
Sum of electronic and thermal Enthalpies =	-1316.467980 (Hartree/Particle)
Sum of electronic and thermal Free Energies =	-1316.539221 (Hartree/Particle)

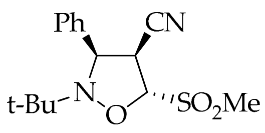

10c

Center	Coordinates (Angstroms) X	Y	Z
C	-0.80984600	-0.79231200	0.38710600
O	-0.83411800	0.47563300	0.96974600
C	0.83789500	0.55406300	-0.63815100
C	-0.12963500	-0.62536200	-0.96877000
H	-0.30188500	-1.52248500	1.02583200
H	1.02726800	1.11874800	-1.55082200
H	-0.86763900	-0.28734200	-1.70095900
N	0.07740700	1.37842400	0.29632700
C	2.15748800	0.09674200	-0.05021800
C	3.20433100	-0.24584200	-0.90274000
C	2.33630000	-0.00848600	1.32691200
C	4.41214700	-0.69787400	-0.38804700
H	3.07528900	-0.16349400	-1.97741400
C	3.54385000	-0.46348300	1.84160100
H	1.53365900	0.28585200	1.99287700
C	4.58312500	-0.81024100	0.98648800
H	5.21961400	-0.96104400	-1.06122600
H	3.67480600	-0.54156300	2.91487900
H	5.52533900	-1.16270000	1.39005300
C	0.50344000	-1.82910300	-1.48076900
N	1.00540700	-2.76917900	-1.90996500
S	-2.53729700	-1.39132900	0.20675100
O	-3.21900900	-0.52750600	-0.74885900
O	-2.45943200	-2.82367900	-0.03637000
C	-3.22432500	-1.09501700	1.82413800
H	-3.17572400	-0.02787500	2.03035400
H	-4.25730600	-1.43925300	1.77901200
H	-2.66007200	-1.67228700	2.55509200
C	-0.65877200	2.55089500	-0.24886300
C	0.42623500	3.58089700	-0.58503800
C	-1.52642000	2.26334200	-1.47957900
C	-1.53137400	3.10052600	0.88187100
H	1.04652900	3.78148400	0.29059300
H	1.07657100	3.24888500	-1.39860400
H	-0.04654200	4.51240700	-0.90417100
H	-2.28418500	1.50720900	-1.26761900
H	-2.04045600	3.17987800	-1.77894500
H	-0.92833500	1.94035500	-2.33761800
H	-1.95301500	4.06153000	0.57897700
H	-2.35499200	2.42269700	1.10828500
H	-0.93804200	3.24750600	1.78710300

Table S27. Thermochemistry and cartesian coordinates of molecule (**11a**) (ωb97xD/6-311G(d,p), in benzene solution (PCM)).

Zero-point correction =	0.251616 (Hartree/Particle)
Thermal correction to Energy =	0.267608 (Hartree/Particle)
Thermal correction to Enthalpy =	0.268552 (Hartree/Particle)
Thermal correction to Gibbs Free Energy =	0.207406 (Hartree/Particle)
Sum of electronic and zero-point Energies =	-1198.628758 (Hartree/Particle)
Sum of electronic and thermal Energies =	-1198.612766 (Hartree/Particle)
Sum of electronic and thermal Enthalpies =	-1198.611822 (Hartree/Particle)
Sum of electronic and thermal Free Energies =	-1198.672968 (Hartree/Particle)

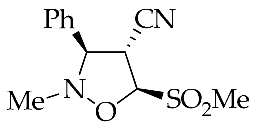

11a

**Center**	**Coordinates (Angstroms)** X	Y	Z
C	-1.55458900	0.22532800	0.18728800
O	-0.95225800	0.21537800	1.46320100
C	0.55397600	1.39710600	0.24017200
C	-0.59661700	0.98689600	-0.74827500
H	-2.56664200	0.63791800	0.20271400
H	0.84386200	2.43430400	0.06242000
H	-0.19801500	0.33308900	-1.52727500
N	-0.04923000	1.31259400	1.56300800
C	1.77242100	0.50820900	0.08227000
C	2.16852200	-0.37927100	1.07467100
C	2.51537600	0.59722500	-1.09358700
C	3.29267000	-1.17549100	0.88845500
H	1.59611100	-0.44156600	1.99095500
C	3.63817400	-0.19647300	-1.27865400
H	2.21734000	1.29387700	-1.87226600
C	4.02842100	-1.08855000	-0.28588100
H	3.59201800	-1.86920200	1.66582900
H	4.20881800	-0.11803200	-2.19677700
H	4.90341400	-1.71216500	-0.42859300
C	-1.23875700	2.12895700	-1.38549600
N	-1.73002500	3.04494000	-1.87712800
S	-1.72532200	-1.51967300	-0.35835000
O	-2.74698300	-1.51473100	-1.39548100
O	-0.39799300	-2.03627700	-0.64928600
C	-2.34941800	-2.33559800	1.09651000
H	-1.61126400	-2.23491500	1.88894200
H	-3.30221100	-1.88580700	1.37194500
H	-2.48455900	-3.37978700	0.81522300
C	-0.80170600	2.50093700	1.95410000
H	-1.28334200	2.29896600	2.90969700
H	-0.08765400	3.31402400	2.08993100
H	-1.56091900	2.81071600	1.22278800

Table S28. Thermochemistry and cartesian coordinates of molecule (**11b**) (ωb97xD/6-311G(d,p), in benzene solution (PCM)).

Zero-point correction =	0.305096 (Hartree/Particle)
Thermal correction to Energy =	0.325101 (Hartree/Particle)
Thermal correction to Enthalpy =	0.326045 (Hartree/Particle)
Thermal correction to Gibbs Free Energy =	0.253572 (Hartree/Particle)
Sum of electronic and zero-point Energies =	-1390.285432 (Hartree/Particle)
Sum of electronic and thermal Energies =	-1390.265427 (Hartree/Particle)
Sum of electronic and thermal Enthalpies =	-1390.264483 (Hartree/Particle)
Sum of electronic and thermal Free Energies =	-1390.336956 (Hartree/Particle)

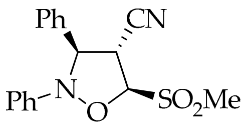

11b

Center	Coordinates (Angstroms) X	Y	Z
C	1.48044700	-0.31456200	0.11980700
O	0.91812100	0.27247000	-1.02453400
C	-0.87880000	-0.06625100	0.42264900
C	0.29316700	-1.05006700	0.73893400
H	1.93906400	0.42327400	0.78941900
H	-1.08780200	0.52850000	1.31515300
H	0.14208900	-1.98869800	0.19805600
N	-0.31917300	0.81949300	-0.61711400
C	-2.13232900	-0.78633900	-0.00946500
C	-2.29462500	-1.20093000	-1.32917300
C	-3.11407600	-1.07788100	0.93249200
C	-3.43219700	-1.90427900	-1.69920900
H	-1.53345200	-0.96002300	-2.06255800
C	-4.25210500	-1.78483500	0.56037800
H	-2.99226500	-0.75020100	1.96041600
C	-4.41177200	-2.19882800	-0.75571500
H	-3.55646400	-2.22175800	-2.72800500
H	-5.01446500	-2.00622000	1.29820400
H	-5.30011600	-2.74638400	-1.04866600
C	0.42690200	-1.33762000	2.15746200
N	0.54681500	-1.56223700	3.27852300
S	2.81327900	-1.41419000	-0.49713000
O	2.18286000	-2.58061900	-1.09383600
O	3.72195400	-0.58528400	-1.26735100
C	3.62705500	-1.90761800	1.01050800
H	4.00059700	-1.02109000	1.52065600
H	2.93556700	-2.47133500	1.63538100
H	4.45784300	-2.54095600	0.69883000
C	-0.21466500	2.19892700	-0.29390500
C	-1.33123400	2.83719600	0.25183700
C	0.93667800	2.93201400	-0.57523800
C	-1.27693100	4.19252400	0.54189600
H	-2.24711500	2.28309200	0.42429600
C	0.97288700	4.28941300	-0.27950900
H	1.78768400	2.44614500	-1.03267300
C	-0.12462900	4.92658700	0.28393000
H	-2.14895500	4.67809300	0.96461900
H	1.87388400	4.85103500	-0.49844500
H	-0.08818300	5.98553100	0.50923900

Table S29. Thermochemistry and cartesian coordinates of molecule (**11c**) (ωb97xD/6-311G(d,p), in benzene solution (PCM)).

Zero-point correction =	0.336012 (Hartree/Particle)
Thermal correction to Energy =	0.357024 (Hartree/Particle)
Thermal correction to Enthalpy =	0.357968 (Hartree/Particle)
Thermal correction to Gibbs Free Energy =	0.284885 (Hartree/Particle)
Sum of electronic and zero-point Energies =	-1316.489404 (Hartree/Particle)
Sum of electronic and thermal Energies =	-1316.468392 (Hartree/Particle)
Sum of electronic and thermal Enthalpies =	-1316.467448 (Hartree/Particle)
Sum of electronic and thermal Free Energies =	-1316.540531 (Hartree/Particle)

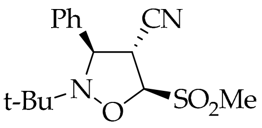

11c

Center	Coordinates (Angstroms) X	Y	Z
C	1.52155400	-0.03722600	0.25788900
O	1.00335500	0.49119200	-0.93049200
C	-0.81644000	0.57216500	0.49564400
C	0.33338500	-0.21945000	1.21188400
H	2.33122300	0.56168300	0.68474400
H	-1.18125100	1.34427300	1.17204300
H	0.05010100	-1.27314900	1.27742200
N	-0.20816400	1.20630600	-0.67811000
C	-1.97514500	-0.32548200	0.12522800
C	-1.88498800	-1.20057900	-0.95594600
C	-3.13738600	-0.29490900	0.88848000
C	-2.94940300	-2.03490300	-1.26345800
H	-0.97755000	-1.23223700	-1.54663800
C	-4.20479100	-1.13166300	0.57910000
H	-3.21395300	0.38864900	1.72872000
C	-4.11157100	-2.00260900	-0.49780400
H	-2.87178300	-2.71567400	-2.10335400
H	-5.10790700	-1.09804200	1.17757600
H	-4.94157200	-2.65551900	-0.74253900
C	0.60874000	0.26740800	2.55607500
N	0.80558100	0.67277300	3.61367500
S	2.25311000	-1.66707500	-0.16288900
O	3.08191000	-2.04288200	0.97294300
O	1.17578900	-2.54262900	-0.59917600
C	3.28392600	-1.29318800	-1.56703100
H	2.65063700	-0.90353600	-2.36127900
H	4.04400500	-0.57397600	-1.26558000
H	3.74424400	-2.23650900	-1.86000900
C	0.04765900	2.67005400	-0.60701300
C	0.70646500	3.06798200	-1.92962900
C	-1.33225000	3.33324300	-0.52224000
C	0.92830000	3.11136300	0.56987500
H	1.68841000	2.60513700	-2.03495300
H	0.08405900	2.75605700	-2.77104300
H	0.83103400	4.15277600	-1.96720100
H	-1.81752300	3.17397800	0.44373000
H	-1.22150600	4.41126400	-0.65658800
H	-1.98720100	2.94777600	-1.30630300
H	1.00618000	4.20109900	0.57873100
H	0.51855500	2.80952000	1.53746600
H	1.94334400	2.71739600	0.47980700
